# CA9‐Targeted PET Imaging for Noninvasive Discrimination of Clear Cell Renal Cell Carcinoma and Associated Tumor Biological Features

**DOI:** 10.1002/advs.76622

**Published:** 2026-07-20

**Authors:** Kailei Chen, Sixuan Cheng, Jian Shi, Ruijie Liu, Yilong Wu, Xin Zheng, Xinlun Song, Yunxuan Zhang, Lei Liu, Zhihao Wei, Qi Wang, Xinwei Li, Zirui Dong, Yuenan Liu, Hongmei Yang, Xiaoli Lan, Chunxia Qin, Dawei Jiang, Keshan Wang, Xiaoping Zhang

**Affiliations:** ^1^ Department of Urology Union Hospital, Tongji Medical College Huazhong University of Science and Technology Wuhan China; ^2^ Institute of Urology Tongji Medical College Huazhong University of Science and Technology Wuhan China; ^3^ Hubei Province Key Laboratory of Precision Radiation Oncology Wuhan China; ^4^ Department of Nuclear Medicine Union Hospital Tongji Medical College Huazhong University of Science and Technology Wuhan China; ^5^ Hubei Key Laboratory of Molecular Imaging Wuhan China; ^6^ Key Laboratory of Biological Targeted Therapy Ministry of Education Wuhan Hubei China; ^7^ Department of Pathogenic Biology School of Basic Medicine Huazhong University of Science and Technology Wuhan China; ^8^ Shenzhen Huazhong University of Science and Technology Research Institute Shenzhen China

**Keywords:** angiogenesis, carbonic anhydrase 9, clear cell renal cell carcinoma, PET imaging, tumor functional states

## Abstract

**Purpose:**

Clear cell renal cell carcinoma (ccRCC) is biologically distinct from non‐clear cell renal cell carcinoma (nccRCC), and shows marked intratumoral heterogeneity, yet current imaging modalities lack the ability to noninvasively distinguish histologic subtypes or capture tumor biological features in vivo.

**Experimental Design:**

An integrative analysis combines CA9‐targeted positron emission tomography (PET) imaging with multi‐omic analyses, histopathological validation, and functional assessment using patient‐derived xenograft (PDX) models. Clinical CA9‐targeted PET imaging is evaluated in patients with renal masses and examined alongside ^18^F‐FDG PET.

**Results:**

CA9 is preferentially enriched in ccRCC tumor cells with low background expression in normal kidney tissue. CA9‐targeted PET imaging shows potential for noninvasive discrimination of ccRCC from nccRCC, including lesions with distinct histologies within individual patients. High CA9‐targeted PET uptake is associated with PBRM1 loss‐of‐function alterations, immune pathway suppression with reduced B cell infiltration, metabolic alterations, and enhanced angiogenic activity. Single‐cell and spatial analyses support endothelial enrichment and angiogenesis‐associated programs in CA9‐high regions. CA9‐high PDX models showed greater tumor growth inhibition under axitinib treatment.

**Conclusions:**

CA9‐targeted PET provides proof‐of‐concept evidence for noninvasive discrimination of ccRCC and associated angiogenic and tumor biological features, warranting prospective validation.

## Graphical abstract

CA9‐targeted PET imaging could be a noninvasive approach to characterize clear cell renal cell carcinoma and associated tumor biology. PET uptake correlates with tumor CA9 expression and is linked to angiogenic activity, immune remodeling, and metabolic reprogramming. Exploratory PDX data further suggest potential relevance for anti‐angiogenic treatment stratification, warranting prospective clinical validation.

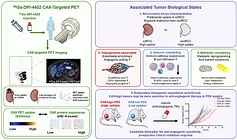

## 1 Introduction

Renal cell carcinoma (RCC) is one of the most common malignancies of the urinary system, with clear cell renal cell carcinoma (ccRCC) accounting for the majority of cases [1, 2]. Given the fundamental differences in molecular background, metabolic characteristics, and therapeutic response between ccRCC and non‐clear cell renal cell carcinoma (nccRCC), accurate discrimination of ccRCC is of critical clinical importance for diagnosis, treatment selection, and prognostic assessment [3, 4]. At present, imaging evaluation of renal tumors primarily relies on anatomy‐based modalities such as computed tomography (CT) and magnetic resonance imaging (MRI). However, these conventional modalities have limited ability to reliably distinguish ccRCC from nccRCC in vivo or to characterize tumor biological features [5, 6, 7]. Accordingly, definitive histological classification still depends on biopsy or surgical specimens as the diagnostic gold standard [2, 8, 9].

Positron emission tomography (PET) offers an important avenue for functional assessment by targeting specific molecular or metabolic processes in vivo [10]. Nevertheless, the most widely used PET tracer in oncology, ^18^F‐fluorodeoxyglucose (FDG), has limited utility in RCC, particularly in ccRCC, due to heterogeneous tumor uptake and high physiological background signal from renal excretion [11, 12]. These limitations underscore the need for more tumor‐specific molecular imaging targets that can capture biological features of ccRCC.

Carbonic anhydrase 9 (CA9) is a transmembrane enzyme highly enriched in ccRCC and is largely absent in most nccRCC subtypes and normal renal parenchyma [13]. Based on this expression pattern, CA9‐targeted molecular imaging strategies have shown encouraging potential for in vivo detection of ccRCC lesions in early clinical studies [14, 15, 16]. However, prior investigations have mainly focused on lesion localization or histological subtype discrimination, with imaging signals primarily interpreted as indicators of tumor presence [10, 14]. Whether CA9 molecular imaging signals reflect broader biological features of ccRCC remains incompletely understood. Specifically, it remains unclear whether CA9‐high ccRCC represents a distinct functional state involving immune, metabolic, and angiogenic programs [17, 18, 19].

In this study, we evaluated CA9‐targeted PET imaging in an exploratory clinical cohort to assess its potential for noninvasive discrimination of ccRCC from nccRCC. We further integrated bulk transcriptomic and untargeted metabolomic analyses with publicly available single‐cell and spatial transcriptomic datasets, together with histological validation and patient‐derived xenograft(PDX)‐based functional assessment, to explore the biological features associated with CA9‐high tumors. We observed that CA9‐high tumors were associated with immune‐suppressive features, constrained metabolic programs, distinct molecular characteristics, and enhanced angiogenic activity, and that CA9‐high PDX models showed greater axitinib‐associated tumor growth inhibition. Together, these findings support CA9‐targeted PET as a promising molecular imaging tool that may aid RCC subtype assessment and provide in vivo biological information related to CA9‐high ccRCC.

## 2 Results

### 2.1 Identification of CA9 as a ccRCC‐Specific Molecular Imaging Target Through Integrated Transcriptomic and Histopathological Analyses

To identify diagnostic biomarkers specific to ccRCC, we first performed differential expression analysis using the Cancer Genome Atlas (TCGA)‐Kidney Renal Clear Cell Carcinoma (KIRC) cohort, comparing tumor tissues with matched adjacent normal kidney tissues (Table S1). Genes significantly upregulated in ccRCC (log_2_ fold change > 2.5, P < 0.001) were intersected with plasma membrane‐associated genes curated from the Molecular Signatures Database (MSigDB) GOCC_PLASMA_MEMBRANE_REGION geneset (Table S2).

To further ensure tumor cell specificity, we incorporated single‐cell RNA sequencing data from the GSE159115 cohort and retained genes predominantly expressed in malignant epithelial cells rather than immune cell populations (tumor expression > 0.5; tumor‐to‐non‐tumor expression ratio > 2; immune cell expression ≤ 0.5) (Table S3). Through this integrative screening strategy, four candidate genes were identified: EGFR, CA9, SLC22A5, and SLC17A3 (Figure 1A). We next evaluated the expression patterns of these candidates across normal human tissues using the Genotype‐Tissue Expression (GTEx) database. EGFR and SLC22A5 exhibited high and widespread expression across multiple normal organs, which would be expected to generate substantial background signal and therefore limit their utility as molecular imaging targets. SLC17A3, although displaying relatively low expression in most extra‐renal tissues, showed high and kidney cortex–enriched expression, resulting in excessive renal background and rendering it unsuitable for renal tumor imaging. In contrast, CA9 displayed minimal expression in normal tissues, including the normal kidney, supporting its potential as a highly specific diagnostic imaging target (Figure 1B and Figure S1).

**FIGURE 1:**
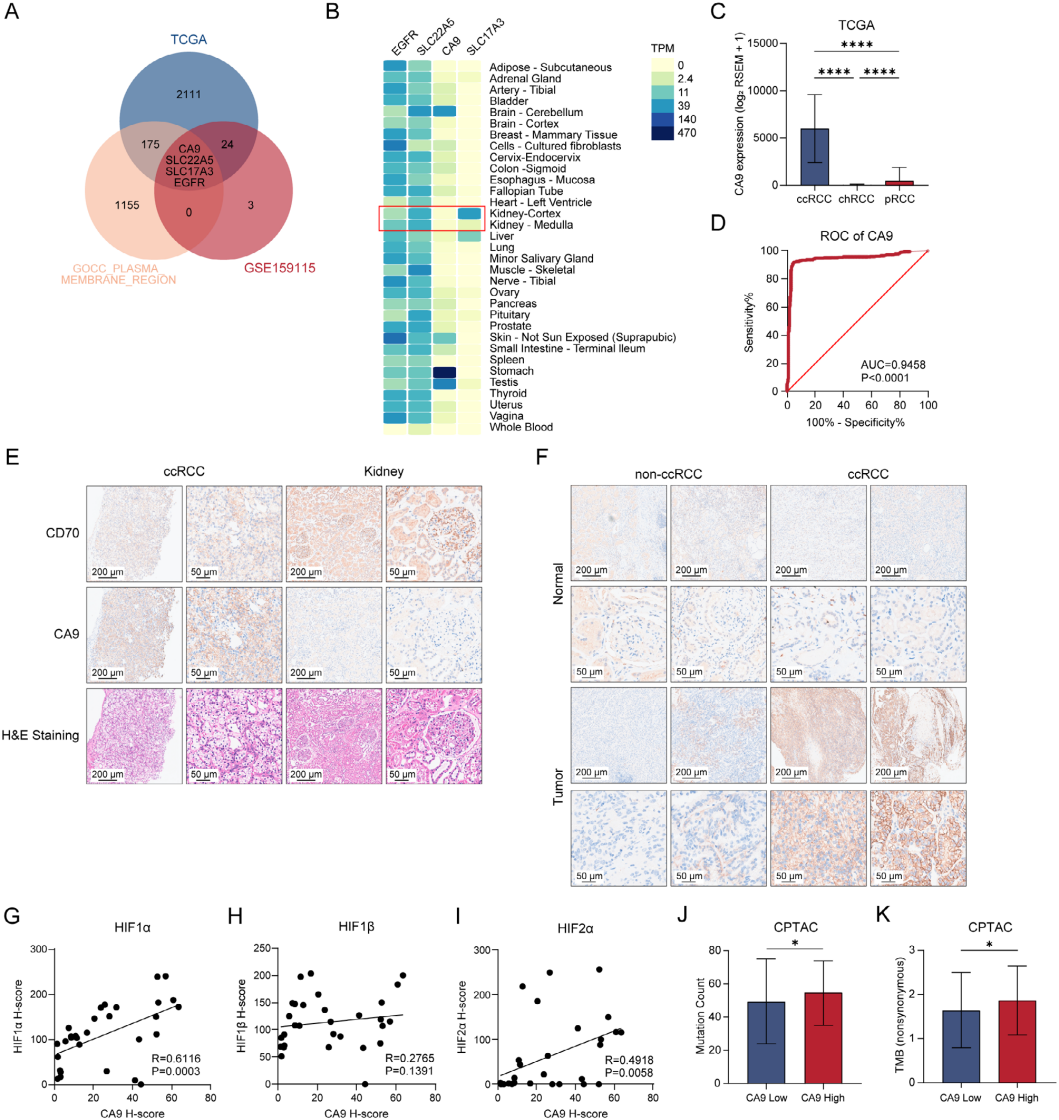
Integrated screening and validation of CA9 as a ccRCC)‐specific diagnostic marker. (A) Venn diagram showing the intersection of genes significantly upregulated in TCGA‐KIRC tumors versus adjacent normal tissues, plasma membrane‐associated genes (GOCC_PLASMA_MEMBRANE_REGION, MSigDB), and genes predominantly expressed in malignant epithelial cells from the GSE159115 single‐cell RNA‐seq dataset. (B) RNA expression levels (TPM) of candidate genes across normal human tissues in the GTEx database. (C) Integrated comparison of CA9 transcriptomic expression across TCGA‐KIRC, TCGA‐KICH, and TCGA‐KIRP cohorts (n = 889). Kruskal‐Wallis test with Dunn's multiple comparisons test. Data are presented as mean ± standard error of the mean (SEM). (D) Receiver operating characteristic (ROC) curve evaluating the diagnostic performance of CA9 in distinguishing ccRCC from nccRCC in the TCGA cohort (n = 889). (E) Representative immunohistochemical (IHC) staining of CA9 and CD70, along with hematoxylin and eosin (H&E) staining, in ccRCC tumors and matched adjacent normal kidney tissues. Scale bars, 50 or 200 µm as indicated. (F) Representative CA9 IHC staining in nccRCC renal tumors and ccRCC samples. Scale bars, 50 or 200 µm as indicated. (G–I) Correlation analysis between CA9 IHC H‐score and protein expression levels of (G) HIF1α, (H) HIF1β, and (I) HIF2α in ccRCC tissues. (Spearman correlation, n = 30) (J) Comparison of total mutation counts between CA9‐low and CA9‐high ccRCC tumors in the CPTAC cohort. Data are presented as mean ± SEM. (Mann‐Whitney U test, n = 352) (K) Comparison of non‐synonymous tumor mutational burden (TMB) between CA9‐low and CA9‐high ccRCC tumors in the CPTAC cohort. Data are presented as mean ± SEM. (Mann‐Whitney U test, n = 352). For panels J and K, CA9‐low and CA9‐high groups were defined using the lower and upper 50% of CA9 expression levels in the CPTAC‐ccRCC cohort, respectively. ^*^ *p* < 0.05; ^**^ *p* < 0.01; ^***^ *p* < 0.001, ^****^ *p* < 0.0001.

Comparative analysis across renal cancer subtypes revealed that CA9 expression was selectively and markedly elevated in ccRCC, while remaining low in chromophobe RCC (chRCC) and papillary RCC (pRCC) (Figure 1C). Consistently, CA9 demonstrated excellent discriminatory performance in distinguishing ccRCC from nccRCC subtypes, with an area under the receiver operating characteristic (ROC) curve (AUC) of 0.9458 (*p* < 0.0001) (Figure 1D).

As CD70 has been previously reported as a ccRCC‐associated membrane marker [20, 21], we performed parallel immunohistochemical (IHC) validation of CA9 and CD70 in renal tumor specimens. While CD70 expression was absent in a subset of ccRCC samples, consistent with previous reports [22], CA9 exhibited robust and specific expression in ccRCC tumors but was undetectable in nccRCC renal cancers and adjacent normal tissues (Figure 1E,F). These findings establish CA9 as a uniquely suitable molecular imaging target for ccRCC, satisfying key requirements including subtype specificity and minimal background expression in normal kidney tissue.

Given that CA9 is a well‐established downstream target of hypoxia signaling, we next assessed the relationship between CA9 expression and hypoxia‐inducible factors in ccRCC tissues. CA9 protein levels were significantly correlated with hypoxia‐inducible factor 1α (HIF1α) and hypoxia‐inducible factor 2α (HIF2α) expression, but not with hypoxia‐inducible factor 1β (HIF1β), consistent with canonical hypoxia‐driven transcriptional regulation (Figure 1G–I).

Finally, analysis of the Clinical Proteomic Tumor Analysis Consortium (CPTAC) cohort revealed that ccRCC tumors with high CA9 expression exhibited significantly increased mutation counts and higher non‐synonymous tumor mutational burden compared with CA9‐low tumors (Figure 1J,K), suggesting that CA9‐high ccRCC represents a genetically more complex tumor subtype.

Together, these results indicate that CA9 uniquely satisfies the key requirements for renal tumor molecular imaging, including low background expression in normal kidney tissue and high subtype specificity for ccRCC.

### 2.2 CA9‐Targeted PET Imaging Reveals High Tumor Uptake and Superior Lesion Conspicuity in ccRCC

To evaluate the in vivo biological and imaging characteristics of CA9 as a molecular imaging target, we employed a previously developed CA9‐targeted PET radiotracer, ^6^ ^8^Ga‐DPI‐4452, for nuclear imaging studies [16]. We first assessed the in vitro safety of DPI‐4452 and found that exposure to the probe did not affect the viability of HK‐2 renal tubular epithelial cells (Figure S2A). We next evaluated tracer uptake in three independent ccRCC patient‐derived xenograft (PDX) models using ^6^ ^8^Ga‐DPI‐4452 PET imaging. All three models exhibited clear tumor uptake, supporting the feasibility of CA9‐targeted PET imaging in vivo (Figure S2B).

We next conducted an exploratory clinical imaging study enrolling a total of 14 patients with renal masses at initial diagnosis or with suspected recurrent ccRCC (Figure 2). Baseline clinical and pathological characteristics of the cohort are summarized in Table 1. The median age was 63 years (range, 26–83 years), and the majority of patients were male (12/14, 85.7%). Most patients had preserved renal function, with only one case of advanced chronic kidney disease. Although limited in size and imbalanced in histologic composition, the cohort included patients with localized and metastatic disease as well as different RCC subtypes, allowing an exploratory assessment of CA9‐targeted PET imaging across clinically heterogeneous cases.

**FIGURE 2:**
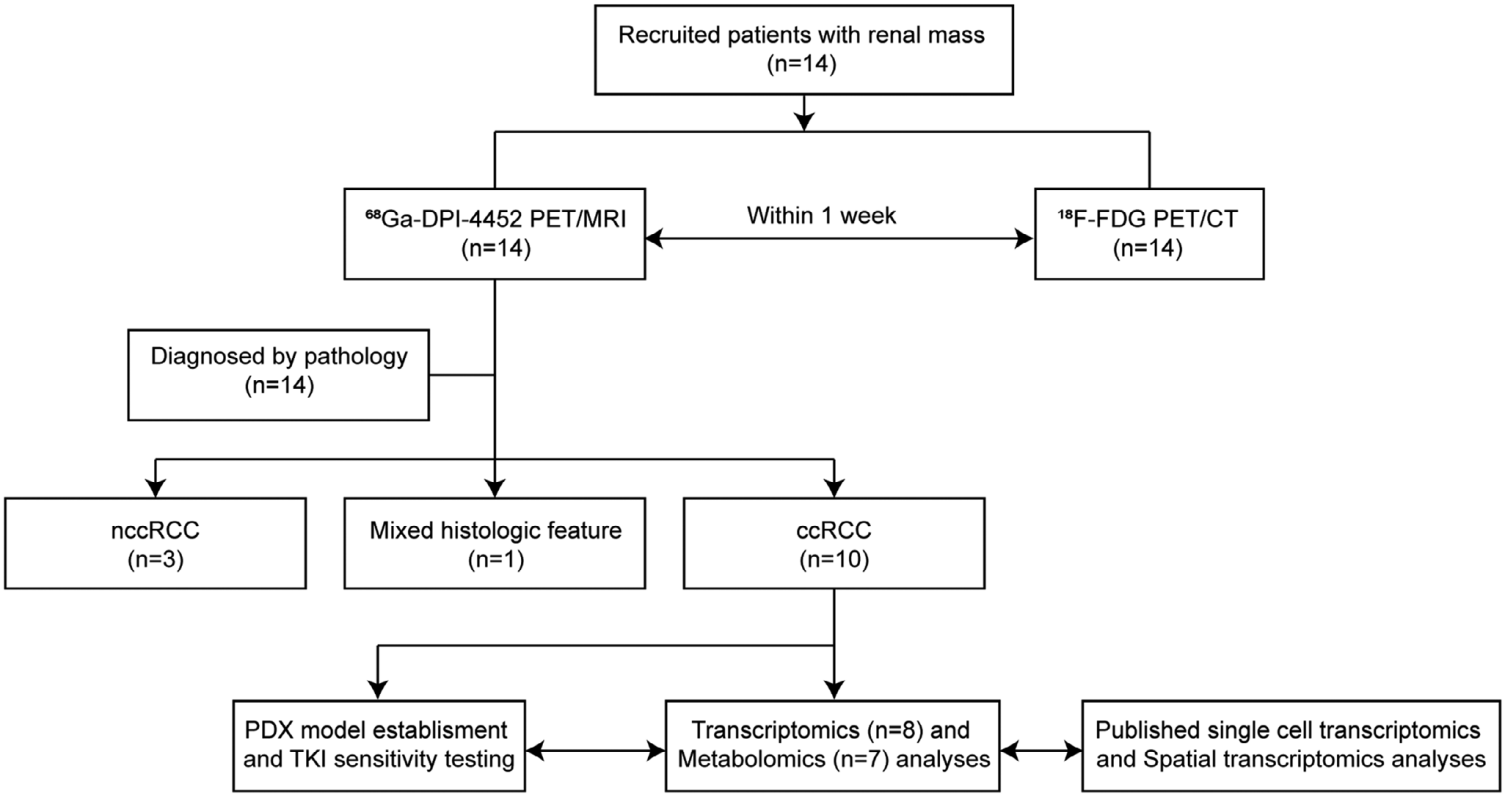
Flow diagram of the clinical study design.

**TABLE 1:** Clinical and pathological characteristics of the study cohort.

Patient ID	Age	Gender	BMI	Cr (µmol/L)	Hb (g/L)	LDH (U/L)	Past medical history	Tumor location	TNM stage	Histologic subtype	ISUP grade	Aggressive pathologic features
1	26	Male	23.66	93.9	144	131	None	Primary renal tumor	T1bN0M0	ccRCC	2/4	Absent
2	66	Male	29.03	86.2	119	158	Hypertension	Multifocal renal tumors	T3bN1M0	Mixed Histology: FH‐deficient RCC + ccRCC	3/4	Renal sinus fat invasion
3	66	Female	24.13	51.4	130	137	None	Primary renal tumor	T1bN0M0	ccRCC	1/4	Absent
4	62	Male	22.86	353.5	97	212	Hypertension, CKD	Primary renal tumor	T1aN0M0	pRCC	2/4	Absent
5	78	Male	19.47	115.2	78	137	Dyslipidemia	Distant metastasis	T1bN0M1	ccRCC	3/4	Absent
6	51	Male	22.65	132.8	85	180	None	Primary renal tumor	T3bN1M0	ccRCC	4/4	Rhabdoid differentiation, Microvascular invasion
7	83	Male	19.86	101.8	102	201	None	Primary renal tumor	T4N1M1	ccRCC	Not available	Absent
8	61	Male	26.30	93.3	89	135	Hypertension, T2DM	Primary renal tumor	T1bN0M0	chRCC	Not applicable	Renal capsule invasion
9	38	Male	20.04	77.6	100	104	None	Primary renal tumor	T1N0M0	Non‐ccRCC, NOS^a^	2/4	Absent
10	60	Male	24.34	116.5	123	130	None	Local recurrence	T1aN0M1	ccRCC	3/4	Absent
11	80	Male	28.65	121.3	102	128	Hypertension, Dyslipidemia	Local recurrence	T1bN0M0	ccRCC	Not available	Not available
12	55	Male	26.31	90.8	121	119	None	Primary renal tumor	T4N1M1	ccRCC	Not available	Not available
13	66	Female	17.78	54.3	140	177	Atrial Fibrillation	Distant metastasis	T1bN0M1	ccRCC	2/4	Absent
14	46	Male	27.78	71.6	164	239	None	Distant metastasis	T1bN0M1	ccRCC	2/4	Absent

^a^ This case was classified as renal cell carcinoma, not otherwise specified (NOS), because definitive histologic subtyping could not be established based on available morphology, and immunohistochemical classification was inconclusive.

**Not applicable** indicates that the corresponding pathological subtype does not support ISUP grading.

**Not available** indicates that ISUP grade or aggressive pathological features could not be evaluated due to insufficient biopsy material.

CA9‐targeted PET imaging demonstrated marked inter‐lesional heterogeneity in tracer uptake across the cohort (Figure 3A), with maximum standardized uptake value (SUVmax) ranging from 6.5 to 171.4 (Table 2). Notably, very high CA9‐targeted PET uptake was observed in several small‐volume lesions, including a 10‐mm local recurrence (SUVmax = 171.4, Patient #10) and a 10‐mm primary renal lesion (SUVmax = 70.3), indicating that CA9‐targeted PET signal intensity was not strictly dependent on lesion size.

**FIGURE 3:**
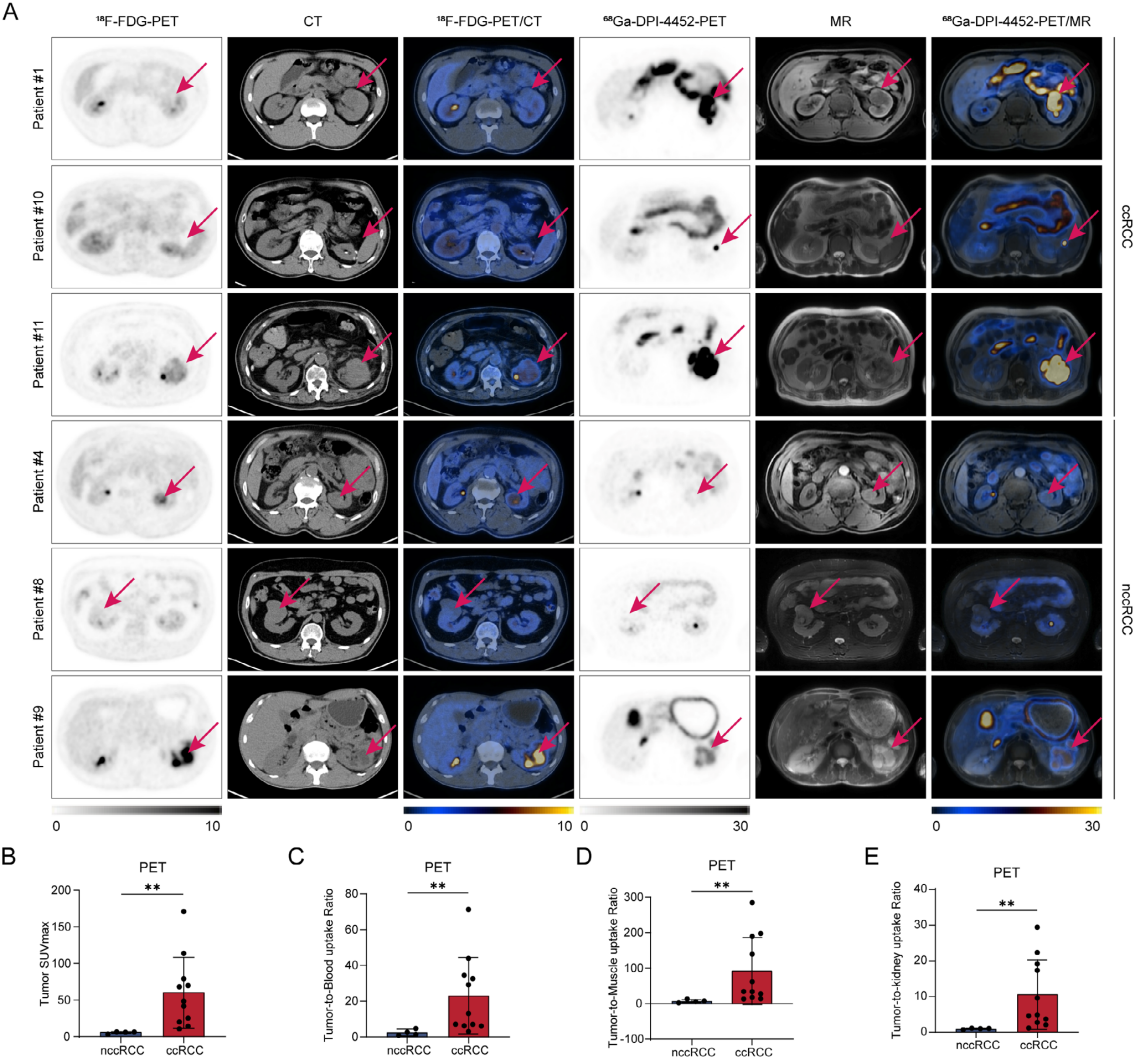
CA9‐targeted PET imaging demonstrates robust tumor uptake and favorable contrast in ccRCC. (A) Representative axial images from enrolled ccRCC patients comparing conventional ^1^ ^8^F‐FDG PET/CT and CA9‐targeted PET/MR acquired 60 min after tracer injection. (B) Maximum standardized uptake values (SUVmax) of ccRCC tumors measured by CA9‐targeted PET/MR in enrolled patients at 60 min post‐injection. Data are presented as mean ± standard deviation. (Mann‐Whitney U test, n = 15) (C) Tumor‐to‐blood uptake ratios derived from CA9‐targeted PET/MR in enrolled patients at 60 min post‐injection. Data are presented as mean ± standard deviation. (Mann‐Whitney U test, n = 15) (D) Tumor‐to‐muscle uptake ratios derived from CA9‐targeted PET/MR in enrolled patients. Data are presented as mean ± standard deviation. (Mann‐Whitney U test, n = 15) (E) Tumor‐to‐kidney uptake ratios derived from CA9‐targeted PET/MR in enrolled patients at 60 min post‐injection. Data are presented as mean ± standard deviation. (Mann‐Whitney U test, n = 15) ^*^ *p* < 0.05; ^**^ *p* < 0.01; ^***^ *p* < 0.001, ^****^ *p* < 0.0001.

**TABLE 2:** PET imaging parameters of index lesions.

Patient ID	Mass largest Diameter (mm)	Index lesion site	SUVmax	TBR‐kidney	TBR‐blood	TBR‐muscle
1	35	Primary renal lesion (left)	79.4	17.26	44.11	198.50
2	60	Primary renal lesion (left, FH deficiency RCC)	7.0	0.95	2.92	14.00
2	10	Primary renal lesion (left, ccRCC)	70.3	9.50	29.29	140.60
3	62	Primary renal lesion (right)	48.9	4.79	13.22	34.93
4	26	Primary renal lesion (left)	3.6	0.62	1.00	3.27
5	22	Right iliac fossa metastasis	68.6	19.06	32.67	62.36
6	76	Primary renal lesion (right)	20.6	2.02	6.24	18.73
7	85	Primary renal lesion (left)	11.1	1.12	3.26	13.88
8	46	Primary renal lesion (right)	6.5	1.00	1.51	6.50
9	40	Primary renal lesion (left)	6.9	1.08	4.93	8.63
10	10	Local recurrence (left)	171.4	22.26	71.42	285.67
11	70	Local recurrence (left)	114.3	29.31	34.64	190.50
12	113	Primary renal lesion (right)	14.5	2.69	7.25	14.50
13	40	Lung metastasis (left)	25.8	3.63	7.17	28.67
14	20	Adrenal gland metastasis (left)	42.0	4.52	6.36	35.00

Consistent with known CA9 expression patterns, lesions with non‐clear cell histology or mixed histologic features including papillary RCC and fumarate hydratase‐deficient RCC (FH‐deficient RCC) exhibited relatively low CA9‐targeted PET uptake (SUVmax approximately 3.6–7.0; Figure 3A, Patients #4, #8, and #9). In contrast, most ccRCC lesions demonstrated moderate to high CA9‐targeted PET uptake, supporting the potential utility of CA9‐targeted imaging for subtype characterization.

Importantly, CA9‐targeted PET imaging showed favorable tumor‐to‐background contrast across lesions, as reflected by elevated tumor‐to‐kidney, tumor‐to‐blood, and tumor‐to‐muscle uptake ratios (Figure 3B–E). These features allowed visualization of both primary renal tumors and metastatic lesions, even in the setting of physiological renal background signal.

As an illustrative comparison, we also examined CA9‐targeted PET imaging alongside conventional ^1^ ^8^F‐FDG PET in selected patients. Consistent with the known limitations of ^1^ ^8^F‐FDG PET in ccRCC [11], several lesions showed low or inconspicuous FDG uptake but clear CA9‐targeted PET signal, including representative lesions in Patients #1, #10, and #11 (Figure 3A). These observations suggest that CA9‐targeted PET may provide complementary lesion visualization in ccRCC, particularly in cases where FDG uptake is limited [11]. Overall, the results support further investigation of CA9 as a potential molecular imaging target for ccRCC in larger prospective cohorts.

### 2.3 Differential CA9‐Targeted PET Uptake in Intrapatient Renal Lesions With Distinct Histologic Subtypes

To further illustrate subtype‐related CA9‐targeted PET uptake at the individual patient level, we analyzed a representative patient (Patient #2) with two spatially distinct renal lesions within the same kidney. Contrast‐enhanced CT revealed dual left renal masses accompanied by renal vein tumor thrombus, raising a high suspicion of malignancy.

On ^1^ ^8^F‐FDG PET/CT, a mass located in the lateral mid‐portion of the left kidney (Lesion 1) exhibited high FDG uptake (SUVmax 12.2–14.1), whereas CA9‐targeted PET/MRI demonstrated no abnormal uptake in this lesion, with signal intensity lower than that of normal renal parenchyma (Figure 4A). In contrast, CA9‐targeted PET imaging identified a second lesion located in the dorsosuperior renal parenchyma (Lesion 2) with marked tracer accumulation (SUVmax 70.3), which was inconspicuous on ^1^ ^8^F‐FDG PET.

**FIGURE 4:**
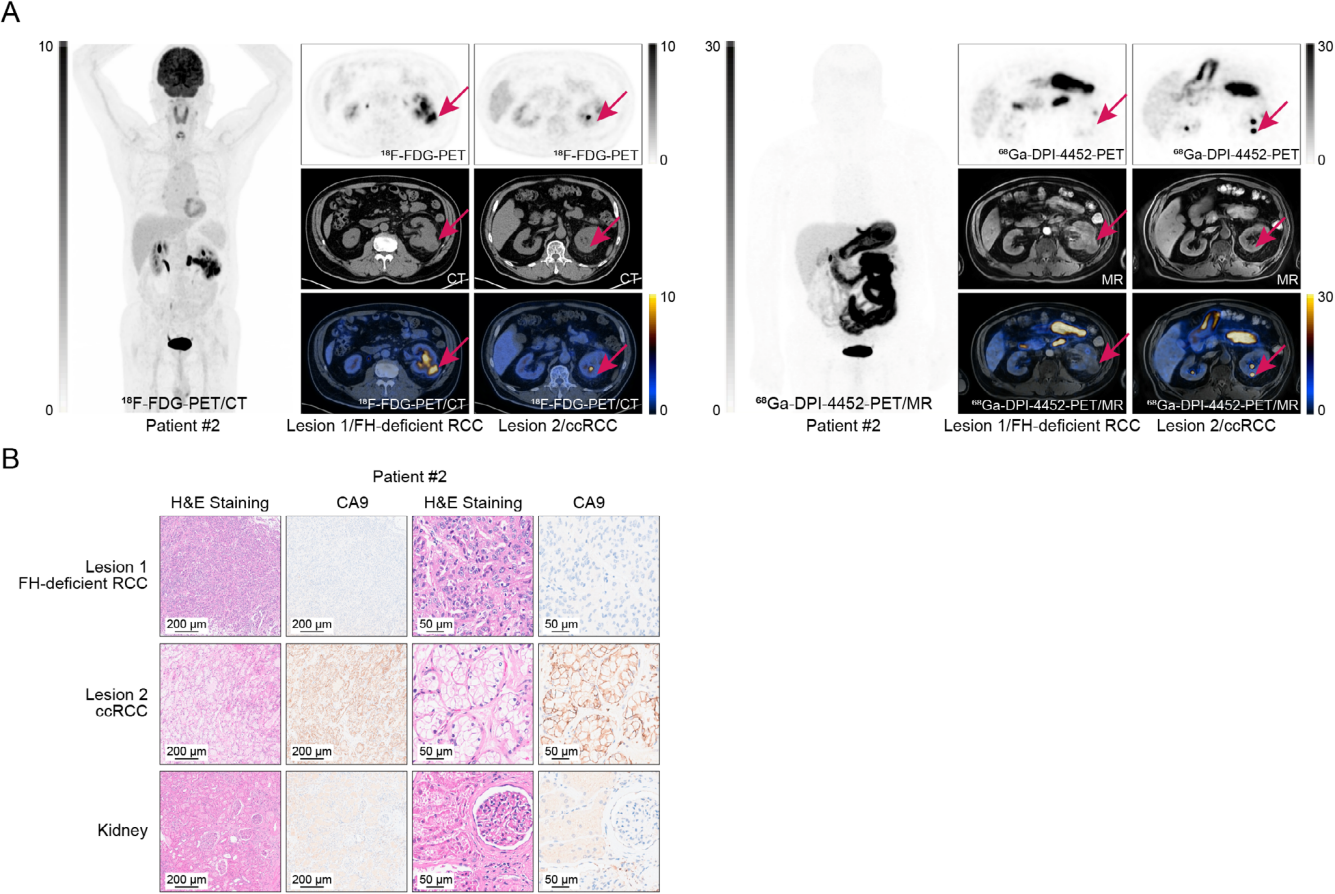
CA9‐targeted PET imaging enables intrapatient discrimination of renal lesions with distinct histologic subtypes. (A) Representative axial images from Patient #2 comparing conventional ^1^ ^8^F‐FDG PET/CT and CA9‐targeted PET/MRI acquired 60 min after tracer injection. Two spatially distinct renal lesions within the left kidney exhibited discordant uptake patterns between FDG and CA9‐targeted PET imaging. (B) Representative hematoxylin and eosin (H&E) staining and CA9 IHC staining of Lesion 1 and Lesion 2 from Patient #2. Scale bars, 50 or 200 µm as indicated.

Postoperative histopathological examination identified Lesion 1 as FH‐deficient RCC and Lesion 2 as ccRCC. Hematoxylin and eosin staining and CA9 IHC staining showed concordant histological and imaging features, with strong CA9 expression observed in the ccRCC lesion but not in the FH‐deficient RCC lesion (Figure 4B).

Together, this representative case suggests that CA9‐targeted PET imaging may help characterize renal lesions with distinct histologic subtypes within the same patient, supporting further investigation of CA9‐targeted PET for molecular characterization and lesion stratification in RCC.

### 2.4 CA9 Expression is Associated With PBRM1 Loss, Genomic Instability, and Dedifferentiation in ccRCC

To investigate whether CA9 expression is associated with aggressive biological features in ccRCC, we first assessed genomic instability at the single‐cell level. Copy number variation (CNV) analysis of single‐cell RNA sequencing (scRNA‐seq) data revealed that tumor cells from CA9‐high tumors exhibited markedly elevated CNV burdens compared with those from CA9‐low tumors, whereas B cells served as non‐malignant controls (Figure 5A). These findings are consistent with our earlier observation that CA9‐high ccRCC patients harbor increased mutation counts and tumor mutational burden at the bulk‐tissue level (Figure 1J–K).

**FIGURE 5:**
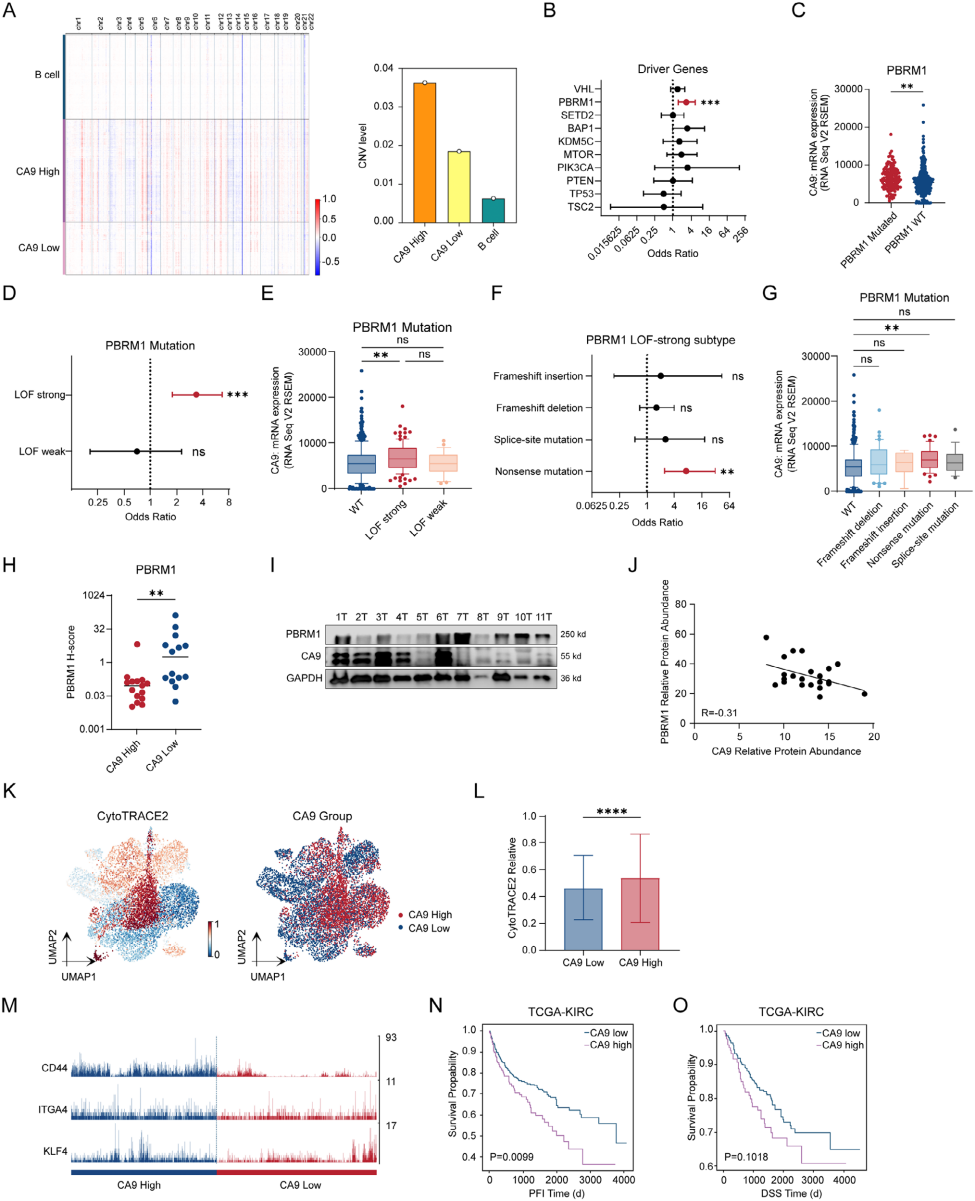
Association of CA9 expression with PBRM1 alterations, tumor cell stemness, and clinical outcome in ccRCC. (A) CNV profiles of tumor cells derived from single‐cell RNA sequencing data, with B cells shown as non‐malignant controls. (B) Odds ratios of mutation frequencies for commonly altered genes in ccRCC comparing CA9‐high and CA9‐low groups, assessed using logistic regression analysis. (C) Comparison of CA9 mRNA expression levels between PBRM1 wild‐type and PBRM1‐mutant ccRCC tumors. Data are presented as mean ± SEM. (Mann–Whitney U test, n = 449) (D) Odds ratios comparing the frequencies of PBRM1 loss‐of‐function (LOF) strong and LOF weak mutations between CA9‐high and CA9‐low ccRCC tumors, analyzed using logistic regression. (E) Differences in CA9 mRNA expression among ccRCC tumors harboring different PBRM1 mutation types. Data are presented as mean ± SEM. Kruskal‐Wallis test with Dunn's multiple comparisons test (n = 449). (F) Odds ratios comparing the frequencies of distinct PBRM1 LOF strong mutation subtypes between CA9‐high and CA9‐low ccRCC tumors, assessed using logistic regression. (G) Comparison of CA9 mRNA expression levels among ccRCC tumors with different PBRM1 LOF strong mutation subtypes. Data are presented as mean ± SEM. Kruskal‐Wallis test with Dunn's multiple comparisons test. (n = 449) (H) IHC H‐scores of PBRM1 in ccRCC tissues stratified by CA9 expression level (CA9‐high, n = 15; CA9‐low, n = 14). (I) Correlation analysis of CA9 and PBRM1 expression levels in ccRCC tissue specimens. Representative images from three independent experiments are shown. (J) Comparison of CA9 and PBRM1 protein abundance in the CPTAC proteomic dataset (PDC000127, n = 20). (K) CytoTRACE2‐derived stemness scores of tumor cells stratified by CA9 expression levels. (L) Comparison of CytoTRACE2 stemness scores between CA9‐high (n = 4365) and CA9‐low (n = 4800) tumor cells. Data are presented as mean ± SEM. (Mann‐Whitney U test) (M) Differential expression of stemness‐associated genes (CD44, ITGA4, and KLF4) between CA9‐high and CA9‐low tumor cells. (N, O) Progression‐free interval (PFI) (N) and disease‐specific survival (DSS) (O) of ccRCC patients from the TCGA‐KIRC cohort stratified by CA9 expression. Stratification criteria: For single‐cell analyses in panels A and K‐M, CA9‐high and CA9‐low tumor cells were defined by median CA9 expression, corresponding to the upper and lower 50%, respectively. For TCGA‐KIRC mutation analyses in panels B, D, and F, CA9‐high and CA9‐low tumors were defined as the upper and lower 25% of CA9 TPM expression among samples with available mutation data. For panel H, tissues were stratified by CA9 IHC H‐score using the upper and lower 50%. For panels N–O, patients were stratified by CA9 expression using the top 25% versus bottom 75%. ^*^ *p* < 0.05; ^**^ *p* < 0.01; ^***^ *p* < 0.001, ^****^ *p* < 0.0001.

We next systematically compared the mutational landscapes of common ccRCC driver genes between CA9‐high and CA9‐low tumors. Among recurrently mutated genes, PBRM1 mutations were significantly enriched in CA9‐high tumors, with an odds ratio (OR) of 2.776 (*p* = 0.001), indicating a strong association between CA9 expression and PBRM1 alteration (Table 3 and Figure 5B). No other canonical ccRCC driver genes showed statistically significant differences between the two groups, although BAP1 mutations displayed a non‐significant trend toward enrichment in CA9‐high tumors (OR = 2.998, *p* = 0.054).

**TABLE 3:** Canonical mutations between CA9‐high and CA9‐low ccRCC.

Gene	Odds ratio	Lower CI	Upper CI	*p*‐value	FDR
VHL	1.422	0.840	2.419	0.206	0.754
PBRM1	2.776	1.482	5.346	0.001	0.010
SETD2	1.000	0.422	2.371	1.000	1.000
BAP1	2.998	0.983	10.965	0.054	0.299
KDM5C	1.635	0.457	6.532	0.571	0.978
MTOR	1.903	0.622	6.470	0.316	0.829
PIK3CA	3.034	0.240	160.941	0.622	0.978
PTEN	1.000	0.224	4.458	1.000	1.000
TP53	0.486	0.104	1.868	0.377	0.829
TSC2	0.497	0.008	9.664	1.000	1.000
TSC1	0.000	0.000	39.001	1.000	1.000

At the transcriptomic level, CA9 mRNA expression was significantly higher in PBRM1‐mutant tumors compared with PBRM1 wild‐type tumors (Figure 5C). Stratification by functional mutation class further revealed that loss‐of‐function (LOF) strong PBRM1 mutations were preferentially enriched in CA9‐high tumors (OR = 3.37, *p* = 0.0002), and tumors harboring LOF strong mutations exhibited significantly elevated CA9 expression relative to wild‐type cases (Figure 5D,E). Among LOF strong mutation subtypes, PBRM1 nonsense mutations showed the most pronounced enrichment in CA9‐high tumors (OR = 7.21, *p* = 0.001; Figure 5E,F).

To evaluate the robustness of this association, we systematically compared the distribution of PBRM1 nonsense mutations across multiple CA9 stratification thresholds. In the CA9 top 50% group, PBRM1 nonsense mutations were observed in 32 of 266 patients, compared with 12 of 266 patients in the CA9 bottom 50% group. This enrichment became more pronounced under stricter stratification criteria: when comparing the top 25% versus the bottom 25% of CA9 expression, PBRM1 nonsense mutations were detected in 19 of 133 CA9‐high tumors but in only 3 of 133 CA9‐low tumors. Under the most stringent 10% versus 10% stratification, PBRM1 nonsense mutations remained detectable exclusively in CA9‐high tumors (6/53), while no such mutations were observed in the corresponding CA9‐low group (0/53) (Table 4). The consistent directionality across stratification strategies supports a stable enrichment of PBRM1 nonsense mutations in CA9‐high ccRCC.

**TABLE 4:** Frequency of PBRM1 truncating mutations across CA9 expression strata.

CA9 expression group	Total, n	Frameshift insertion, n (%)	Frameshift deletions, n (%)	Splice‐site mutation, n (%)	Nonsense mutations, n (%)
Top 10%	53	0 (0%)	5 (9.43%)	1 (1.88%)	6 (11.32%)
Bottom 10%	53	1 (1.9%)	4 (7.55%)	0 (0%)	0 (0%)
Top 25%	133	2 (1.5%)	14 (10.53%)	5 (3.76%)	19 (14.28%)
Bottom 25%	133	1 (0.75%)	9 (6.76%)	1 (0.75%)	3 (2.26%)
Top 50%	266	5 (1.88%)	22 (8.27%)	10 (3.76%)	32 (12.03%)
Bottom 50%	266	3 (1.13%)	18 (6.77%)	5 (1.88%)	12 (4.51%)

At the protein level, IHC, western blotting, and CPTAC‐based proteomic analyses consistently demonstrated reduced PBRM1 protein abundance in CA9‐high tumors (Figure 5H–J and Figure S3A), further supporting a functional link between elevated CA9 expression and PBRM1 loss.

Given that VHL is the most frequently altered gene in ccRCC, we additionally examined whether VHL mutation status was associated with CA9 expression levels. However, no significant difference in VHL mutation frequency was observed between CA9‐high and CA9‐low tumors across subtype analyses (Figure S3B–E).

Given the established role of PBRM1 in chromatin regulation and cellular differentiation, we next examined the differentiation status of CA9‐high tumor cells. CytoTRACE2 analysis revealed significantly higher stemness scores in CA9‐high tumor cells, accompanied by upregulation of multiple stemness‐associated genes, including CD44, ITGA6, and KLF4 (Figure 5K–M).

Finally, survival analysis in the TCGA‐KIRC cohort demonstrated that patients with CA9 expression in the top 25% experienced significantly worse progression‐free interval compared with the remaining patients (Figure 5N), linking CA9‐associated molecular features to adverse clinical outcomes.

### 2.5 CA9‐High ccRCC is Characterized by Immune Suppression and Coordinated Metabolic Reprogramming

To further investigate whether CA9 expression is associated with additional biological features in ccRCC, we performed integrative transcriptomic and metabolomic analyses stratified by CA9 expression levels.

Gene Ontology (GO) enrichment analysis of differentially expressed genes between CA9‐high and CA9‐low tumors in the TCGA‐KIRC cohort identified several immune‐related biological processes among the altered transcriptional programs, including complement activation, humoral immune response mediated by circulating immunoglobulin, acute‐phase response, and B cell‐mediated immunity (Figure 6A–C). These results indicate immune pathway remodeling rather than necessarily immune activation. In parallel, Kyoto Encyclopedia of Genes and Genomes (KEGG) pathway analysis of RNA sequencing (RNA‐seq) data from the institutional ccRCC cohort stratified by CA9‐targeted PET uptake levels similarly highlighted alterations of immune‐associated pathways, including transforming growth factor‐β (TGF‐β) signaling (Figure 6D). Gene set enrichment analysis (GSEA) demonstrated global suppression of immune‐related pathways in CA9‐high tumors (Figure 6E). Consistently, immune cell deconvolution analysis revealed significantly reduced B cell and naïve B cell infiltration in CA9‐high tumors (Figure 6F,G). Moreover, tumors from patients achieving stable disease (SD) following immune checkpoint blockade exhibited higher CA9 expression compared with those achieving partial response (PR) (Figure 6H). Collectively, these findings suggest that CA9‐high tumors exhibit immune pathway remodeling with an overall immunosuppressive pattern, particularly involving reduced B cell‐associated immunity.

**FIGURE 6:**
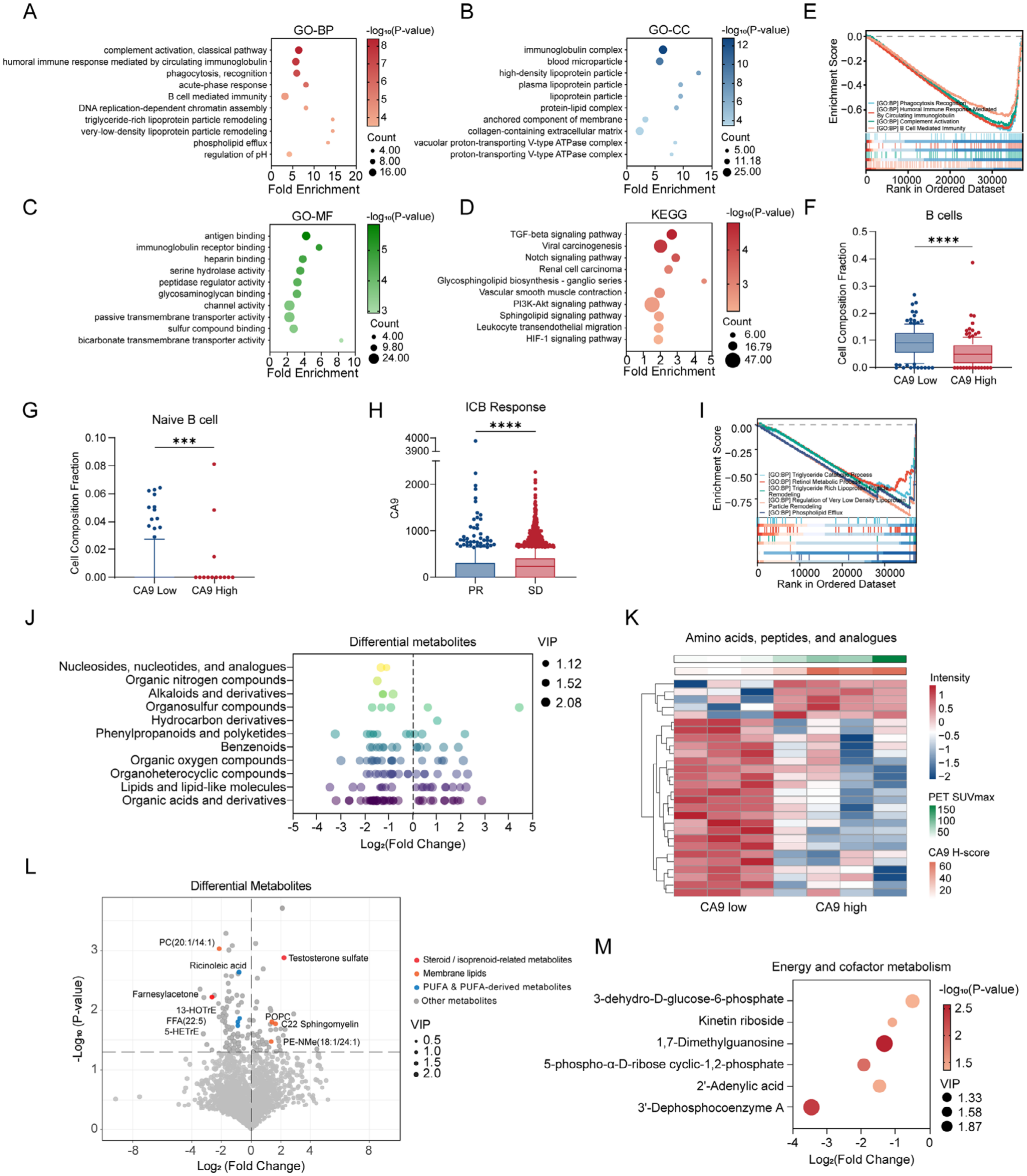
CA9‐high ccRCC exhibits coordinated immune suppression and metabolic reprogramming. (A‐C) Gene Ontology (GO) enrichment analyses of differentially expressed genes between CA9‐high and CA9‐low tumors in the TCGA‐KIRC cohort, showing representative enriched terms for biological process (BP) (A), cellular component (CC) (B), and molecular function (MF) (C). (D) Kyoto Encyclopedia of Genes and Genomes (KEGG) pathway enrichment analysis of RNA‐seq data from the institutional ccRCC cohort stratified by median CA9‐targeted PET SUVmax. (E) Gene set enrichment analysis (GSEA) comparing immune‐related pathways between CA9‐high and CA9‐low tumors in the TCGA‐KIRC cohort. (F, G) xCell‐based estimation of immune cell infiltration levels, showing relative abundance of B cells (F) and naïve B cells (G) in CA9‐high (n = 133) and CA9‐low (n = 133) ccRCC tumors. Data are presented as mean ± SEM. (Mann‐Whitney U test) (H) Comparison of CA9 expression levels between immune checkpoint blockade (ICB)–treated ccRCC patients achieving partial response (PR, n = 447) or stable disease (SD, n = 3066). Data are presented as mean ± SEM. (Mann‐Whitney U test) (I) GSEA comparing metabolism‐related pathways between CA9‐high and CA9‐low ccRCC tumors in the TCGA‐KIRC cohort. (J) Classification of differentially abundant metabolites identified by untargeted metabolomic profiling of ccRCC specimens stratified by CA9‐targeted PET uptake levels. (K) Heatmap of differentially abundant amino acid‐related metabolites detected by untargeted metabolomics in ccRCC specimens stratified by high versus low CA9‐targeted PET uptake. (L) Volcano plot showing significantly altered metabolites identified by untargeted metabolomic analysis of ccRCC specimens grouped by CA9‐targeted PET uptake levels. (M) Differential abundance of metabolites involved in energy and cofactor metabolism identified by untargeted metabolomics in ccRCC specimens stratified by CA9‐targeted PET uptake. Stratification criteria: For transcriptomic analyses in panels A–C, E and I, CA9‐high and CA9‐low tumors were defined by median CA9 expression, corresponding to the upper and lower 50%, respectively. For the institutional RNA‐seq analysis in panel D, tumors were stratified by median CA9‐targeted PET SUVmax, corresponding to the upper and lower 50%, respectively. For xCell immune infiltration analyses in panels F, G, CA9‐high and CA9‐low tumors were defined as the upper and lower 25% of CA9 expression, respectively. For metabolomic analyses in panels J‐M, ccRCC specimens were stratified by median CA9‐targeted PET SUVmax, corresponding to the upper and lower 50%, respectively. ^*^ *p* < 0.05; ^**^ *p* < 0.01; ^***^ *p* < 0.001, ^****^ *p* < 0.0001.

Beyond immune alterations, CA9‐high tumors also displayed pronounced metabolic differences. GO and KEGG analyses revealed enrichment of pathways related to triglyceride‐rich lipoprotein remodeling, very‐low‐density lipoprotein (VLDL) particle remodeling, and regulation of intracellular pH (Figure 6A–D,I), suggesting broad metabolic alterations associated with CA9 expression.

Untargeted metabolomic profiling of ccRCC specimens stratified by CA9‐targeted PET uptake identified a set of significantly altered metabolites (variable importance in projection [VIP] > 1, *p* < 0.05), revealing coordinated metabolic changes dominated by amino acid and lipid metabolism in CA9‐high tumors (Figure 6J). Metabolites involved in amino acid recycling and peptide turnover exhibited a consistent decrease, including γ‐glutamyl cycle intermediates (γ‐glutamylglutamine, γ‐glutamylglutamate, pyroglutamic acid, and glutamate), together with a broad depletion of dipeptides and tripeptides (Figure 6K).

Lipid metabolic alterations were also prominent in CA9‐high ccRCC. Multiple polyunsaturated fatty acids (PUFAs) and their oxidized derivatives, such as 5‐hydroxyeicosatrienoic acid (5‐HETrE), FFA(22:5), ricinoleic acid, and 13‐hydroxy‐octadecatrienoic acid (13‐HOTrE), were significantly reduced (Figure 6L). In contrast, several membrane‐associated lipid species were increased, including POPC, PE‐NMe(18:1_24:1), and C22 sphingomyelin, while specific phosphatidylcholine species such as PC(20:1_14:1) were decreased, indicating alterations in lipid composition. In addition, metabolites related to alternative lipid catabolic and steroid metabolic pathways, including dodecanedioic acid, testosterone sulfate, and farnesyl acetone, were differentially altered.

In parallel, metabolites related to energy metabolism and metabolic cofactors exhibited a coordinated decrease in CA9‐high tumors, including 3′‐dephosphocoenzyme A, adenylic acid (AMP), 5‐phospho‐α‐D‐ribose cyclic‐1,2‐phosphate, modified nucleosides (1,7‐dimethylguanosine and kinetin riboside), and the glycolytic intermediate 3‐dehydro‐D‐glucose‐6‐phosphate (Figure 6M).

Taken together, CA9‐high ccRCC exhibits coordinated alterations in immune‐related pathways and multiple metabolic processes, including amino acid, lipid, and energy metabolism.

### 2.6 CA9‐Targeted PET Uptake is Associated With Angiogenic Activity

Given that transcriptomic analyses revealed enrichment of angiogenesis‐related pathways in CA9‐high tumors (Figure 6D), and considering the central role of anti‐angiogenic tyrosine kinase inhibitors (TKIs) in advanced RCC, we next investigated whether CA9 molecular imaging signals were associated with tumor angiogenic activity. We first examined the biological relevance of CA9‐targeted PET imaging by assessing its relationship with CA9 expression and hypoxia‐related transcriptional programs (Figure S4A–C).

In ccRCC patients undergoing CA9‐targeted PET imaging, tumor CA9‐targeted PET uptake (SUVmax) showed a significant positive association with angiogenesis pathway activity at the transcriptomic level (Figure 7A,B). Secondary analysis of publicly available scRNA‐seq datasets further demonstrated an increased proportion of endothelial cells in CA9‐high tumors (Figure 7C–E). To further investigate the spatial relationship between CA9 expression and angiogenic activity, we reanalyzed the publicly available spatial transcriptomic dataset GSE175540, specifically samples GSM5924052 and GSM5924046 [23]. This analysis revealed pronounced spatial co‐localization between CA9 expression and angiogenesis‐related gene signatures (Figure 7F,G). At the histological level, CA9‐high ccRCC specimens exhibited increased expression of VEGFA, VEGFR2, ANGPT2, and CD31 compared with CA9‐low tumors, further supporting enhanced angiogenic activity and increased vascularity in CA9‐high tumors (Figure 7H,I and Figure S4D–F).

**FIGURE 7:**
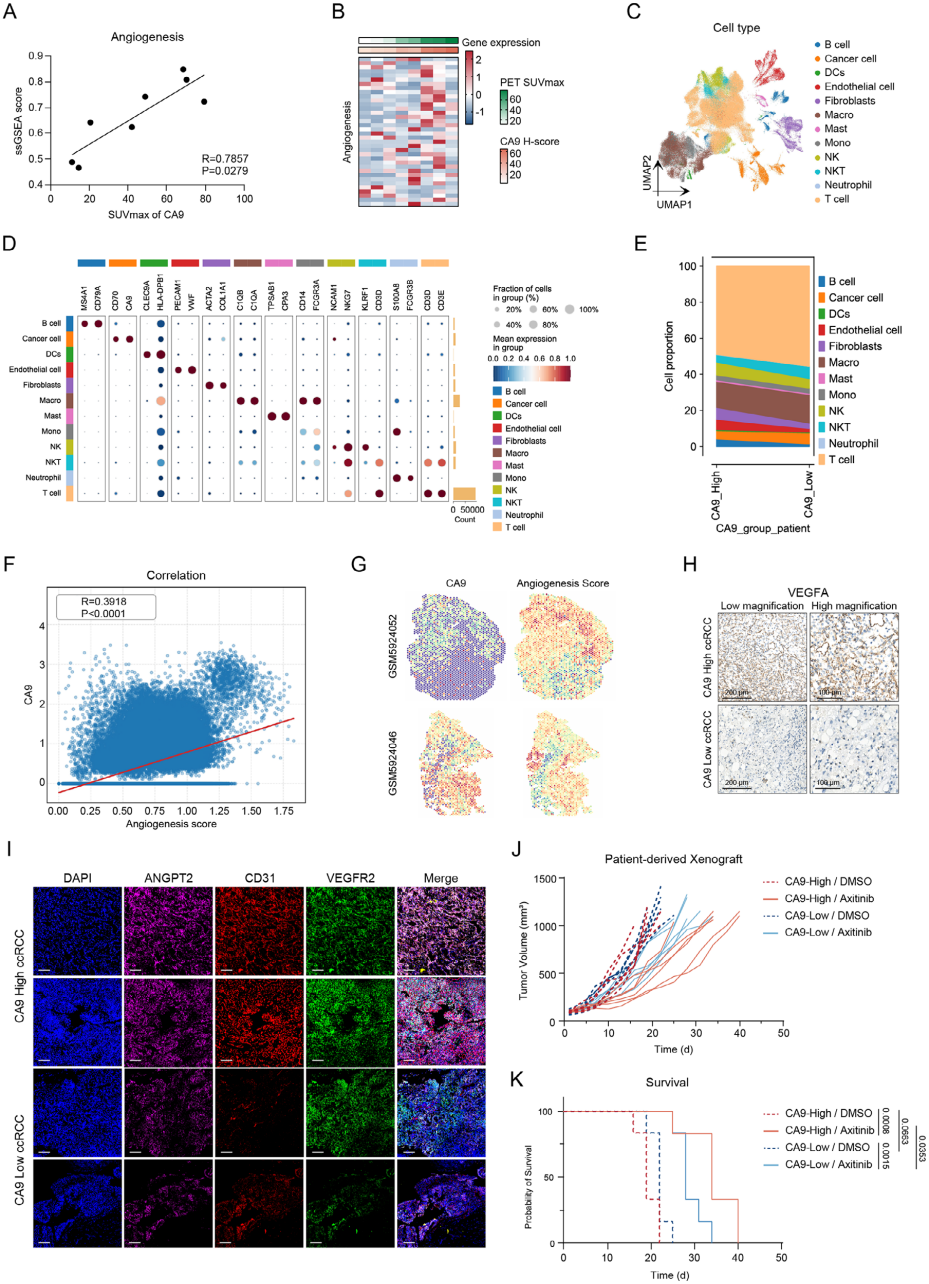
CA9‐targeted PET signal is associated with angiogenic activity in ccRCC. (A) Correlation analysis between CA9‐targeted PET SUVmax values and angiogenesis scores in ccRCC patients. Spearman correlation (n = 8). (B) Heatmap showing CA9‐targeted PET SUVmax, CA9 IHC H‐scores, and expression levels of angiogenesis‐related genes in ccRCC tumor samples. (C) Cell clustering from publicly available scRNA‐seq data of ccRCC tumors. (D) Annotated cell types identified by publicly available scRNA‐seq and their representative marker genes. (E) Comparison of cell‐type‐specific gene expression profiles between CA9‐high and CA9‐low ccRCC tumors. (F) Correlation analysis between CA9 expression and angiogenesis scores in publicly available ccRCC spatial transcriptomic data GSE175540. (Spearman correlation) (G) Spatial distribution of CA9 expression and angiogenesis scores in ccRCC tissue sections from the publicly available spatial transcriptomics dataset GSE175540. The spatial transcriptomics data of samples GSM5924052 and GSM5924046 were reanalyzed in this study. (H) Representative immunohistochemical staining of VEGFA in CA9‐high and CA9‐low ccRCC tumor specimens (scale bar in low‐magnification field = 200 µm, scale bar in low‐magnification field = 100 µm). (I) Representative immunofluorescent staining of CD31, VEGFR2, and ANGPT2 in CA9‐high and CA9‐low ccRCC tumor specimens (scale bar = 200 µm). (J, K) Tumor growth curves (J) and survival analysis (K) of CA9‐high and CA9‐low ccRCC PDX models treated with DMSO or the anti‐angiogenic agent axitinib (n = 6 for each group). Stratification criteria: For the scRNA‐seq analysis in panels C–E, CA9‐high and CA9‐low tumors were defined by median CA9 expression, corresponding to the upper and lower 50%, respectively. For panel H and I, representative images were selected from two tumors in the upper 50% and two tumors in the lower 50% of CA9‐targeted PET SUVmax values. For panels J‐K, CA9‐high and CA9‐low PDX models were defined according to relative CA9 protein expression levels confirmed by Western blotting in PDX tumor tissues.

We next assessed whether the angiogenic features associated with CA9 expression were observed in PDX models. Consistent with the clinical specimens, CA9‐high PDX tumors exhibited significantly increased CD31‐positive areas compared with CA9‐low PDX tumors, indicating increased vascularity in the CA9‐high models (Figure S4G–I).

We further evaluated the relationship between CA9 expression and response to anti‐angiogenic therapy. In PDX models established from CA9‐high and CA9‐low ccRCC tumors, axitinib treatment was associated with greater tumor growth inhibition and prolonged survival in CA9‐high models compared with CA9‐low models (Figure 7J,K and Figure S4J). In line with our observations, prior experimental studies have reported induction of CA9 expression following vascular endothelial growth factor (VEGF)‐targeted therapy and reduced antiproliferative efficacy of sunitinib upon CA9 silencing in ccRCC models [24].

Together, these findings indicate that CA9‐targeted PET uptake is associated with angiogenic activity across multiple molecular and histological levels. The exploratory PDX results further suggest a potential association between CA9‐high angiogenic features and axitinib response, warranting further prospective studies to determine whether CA9‐targeted PET may help inform anti‐angiogenic treatment stratification in ccRCC.

## 3 Discussion

In this study, we explored the biological significance of CA9‐targeted molecular imaging in ccRCC, examining whether its value may extend beyond lesion visualization toward biologically informative imaging. By integrating transcriptomic screening, histopathological validation, and clinical CA9‐targeted PET imaging, we found that CA9‐targeted PET may help noninvasive discrimination of ccRCC from nccRCC, and that its uptake is also associated with tumor biological features in ccRCC. These findings support further evaluation of CA9‐targeted PET as a molecular imaging approach with potential biological informativeness [10, 14].

One important aspect of this work is the biological rationale for CA9 as a particularly relevant molecular imaging target for ccRCC. Although CA9 has long been recognized as a ccRCC‐associated marker, prior studies largely relied on its known expression pattern without addressing why CA9 may be advantageous compared with other membrane‐associated candidates from an imaging perspective. Through integrated analyses of bulk transcriptomics, curated membrane protein annotations, single‐cell RNA sequencing, and normal tissue expression profiling, we found that CA9 fulfilled the combined requirements of tumor cell enrichment, subtype selectivity, and low physiological background in normal kidney tissue. This analysis provides a biological rationale for the observed performance of CA9‐based imaging and helps distinguish CA9 from membrane proteins limited by broad expression or excessive renal background.

Clinical imaging results further indicate that CA9‐targeted PET uptake may reflect biologically meaningful tumor contrast rather than lesion presence or anatomical size alone. ^18^F‐FDG PET showed low or heterogeneous uptake in ccRCC lesions, whereas CA9‐targeted PET showed favorable tumor‐to‐background contrast in multiple lesions, even within the physiologically tracer‐avid renal environment. Importantly, CA9‐targeted PET signal intensity was not strictly dependent on lesion volume, suggesting that tracer uptake may primarily reflect intrinsic molecular features rather than anatomical parameters. This characteristic may differentiate CA9‐targeted PET from conventional metabolic imaging and supports its further investigation as a ccRCC‐associated functional imaging approach.

The clinical relevance of CA9‐targeted PET‐based subtype discrimination is illustrated by complex intrapatient scenarios. In a representative patient harboring both ccRCC and FH‐deficient RCC within the same kidney, CA9‐targeted PET showed differential uptake between lesions that were indistinguishable by anatomical imaging and exhibited discordant FDG uptake. The concordance between CA9‐targeted PET signal and CA9 IHC expression further supports subtype‐related molecular specificity of this imaging strategy at the individual lesion level. Although based on a single illustrative case, this intrapatient observation highlights the potential utility of CA9‐targeted PET in multifocal or histologically heterogeneous renal tumors, where biopsy sampling may be limited or biased.

Several prior clinical studies, most notably the multicenter phase III trial of ^89^Zr‐girentuximab PET/CT, have established the feasibility and diagnostic accuracy of CA9‐targeted imaging for ccRCC detection [15]. Our study builds on these efforts by exploring the biological interpretation of CA9‐targeted PET uptake, suggesting that this imaging signal may reflect coordinated tumor‐intrinsic programs rather than serving solely as an isolated molecular marker.

Beyond diagnostic discrimination, our integrative analyses suggest that CA9‐high ccRCC may represent a biologically coherent tumor phenotype. At the genomic and epigenetic levels, these tumors exhibit increased CNV and enrichment of PBRM1 LOF mutations with reduced protein expression, consistent with chromatin dysregulation. Single‐cell analyses further revealed elevated CytoTRACE stemness scores and activation of stemness‐associated programs, collectively suggesting that CA9‐high tumors may be associated with a genomically unstable and poorly differentiated tumor state.

This CA9‐high tumor phenotype was also associated with immune pathway remodeling and an overall immunosuppressive pattern. Transcriptomic analyses identified immune‐related pathways among differentially expressed genes, but these findings should not be interpreted as evidence of immune activation. Instead, GSEA revealed suppression of multiple immune pathways, including B cell‐mediated immunity, complement activation, and humoral immune responses. Immune deconvolution analyses showed reduced infiltration of B cells and naïve B cells in CA9‐high tumors [25]. Moreover, among patients treated with immune checkpoint blockade, those achieving SD exhibited higher CA9 expression than those with PR, supporting the hypothesis that CA9‐high tumors may reside within an immunologically suppressed microenvironment with limited responsiveness to immunotherapy.

Metabolomic profiling further suggested that CA9‐high ccRCC tumors undergo metabolic constraint rather than isolated pathway activation. Widespread depletion of dipeptides, tripeptides, and metabolites involved in amino acid recycling and the γ‐glutamyl cycle was consistent with sustained metabolic stress. In parallel, suppression of PUFAs and their oxidized derivatives, selective remodeling of membrane‐associated lipids, and uniform reductions in metabolites related to energy production, nucleotide turnover, and metabolic cofactors collectively suggested a metabolic core. These findings support the notion that CA9‐high ccRCC may represent a metabolically restricted, rather than compensatorily activated, tumor state.

Against this background of immune suppression and metabolic constraint, CA9‐high tumors exhibited features associated with increased angiogenic activity. CA9‐targeted PET uptake correlated with angiogenesis‐related transcriptional signatures, endothelial cell abundance, and spatial co‐localization of angiogenic scores in spatial transcriptomic analyses. Histologically, CA9‐high tumors displayed increased microvessel density. In exploratory PDX models, tumors established from CA9‐high specimens showed greater tumor growth inhibition and prolonged survival following treatment with the anti‐angiogenic TKI axitinib compared with CA9‐low models. These results suggest a potential association between CA9‐targeted PET‐associated tumor states and axitinib response in preclinical models. Notably, this observation is consistent with previous experimental evidence supporting a functional interplay between CA9 expression and angiogenic dependency under VEGF‐targeted therapy [24]. However, whether CA9‐targeted PET can reliably predict clinical response to anti‐angiogenic therapy requires prospective validation.

Several limitations should be acknowledged. The clinical CA9‐targeted PET cohort was modest in size and exploratory in nature, precluding definitive conclusions regarding diagnostic performance or predictive accuracy. Future prospective studies with larger, ideally multicenter cohorts that include patients across different TNM stages, localized and metastatic disease states, and early‐ to advanced‐stage ccRCC are needed to validate the diagnostic and biological implications of CA9‐targeted PET in more clinically heterogeneous settings. In addition, our analyses primarily establish associations between CA9‐targeted PET uptake and tumor biological features rather than direct molecular causality. Further mechanistic studies are required. Finally, exploration of therapeutic sensitivity was limited to PDX models and warrants confirmation in prospective clinical studies.

## 4 Conclusion

This study suggests that CA9‐targeted PET may provide an imaging‐based approach for understanding biological heterogeneity in ccRCC. CA9‐targeted PET uptake was associated with hypoxia‐adapted, angiogenesis‐associated, immunosuppressed, and metabolically constrained tumor features, supporting further evaluation of this approach for biological stratification and prospective therapeutic assessment in RCC.

## 5 Experimental Section

### 5.1 Human RCC Specimens and Clinical Study Registration

Matched RCC tissues and adjacent non‐tumorous renal tissues were obtained from patients undergoing surgical resection at Union Hospital, Tongji Medical College, Huazhong University of Science and Technology. All specimens were snap‐frozen in liquid nitrogen immediately after excision and stored at −80 °C until further use. Written informed consent was obtained from all participants prior to surgery. This clinical study was registered both domestically and internationally (China Primary Registry Identifier: 273914; ClinicalTrials.gov identifier: NCT07301827).

### 5.2 Cell Viability Assay

Cell viability was assessed using a Cell Counting Kit‐8 (CCK‐8) assay. Non‐radioactive DPI‐4452 was purchased from MedChemExpress (HY‐P10761). Cells were seeded in 96‐well plates at 4000 cells per well and treated with dimethyl sulfoxide (DMSO) or DPI‐4452 at the indicated concentrations for 48 h. CCK‐8 reagent (MedChemExpress, HY‐K0301) was then added to each well, and absorbance at 450 nm was measured using a microplate reader after incubation at 37 °C for 1.5 h. Cell viability was normalized to vehicle‐treated controls after background subtraction. Each condition included four technical replicates and was repeated in three independent experiments.

### 5.3 Cell Culture

Human renal proximal tubular epithelial HK‐2 cells were obtained from American Type Culture Collection and cultured in Dulbecco's modified Eagle's medium/F12 (DMEM/F12; Servicebio, Wuhan, China) supplemented with 10% fetal bovine serum (Vazyme, Nanjing, China) and 1% penicillin‐streptomycin. Cells were maintained at 37 °C in a humidified atmosphere with 5% CO_2_. All cells were routinely confirmed to be free of mycoplasma contamination.

### 5.4 Tissue Microarray Construction and Immunohistochemistry

Tissue microarrays (TMAs) were constructed from formalin‐fixed, paraffin‐embedded (FFPE) human RCC specimens. IHC staining was performed using standard protocols. Briefly, paraffin sections were deparaffinized, rehydrated, and subjected to antigen retrieval using either citrate buffer (0.01 M, pH 6.0) or ethylenediaminetetraacetic acid (EDTA) buffer (0.01 M, pH 9.0), depending on the antibody. Endogenous peroxidase activity was quenched with 3% hydrogen peroxide for 30 min.

Sections were incubated with primary antibodies at 4°C overnight, followed by incubation with horseradish peroxidase (HRP)‐conjugated secondary antibodies at 37 °C for 45 min. Signal detection was performed using 3,3′‐diaminobenzidine (DAB) substrate, and nuclei were counterstained with hematoxylin. Hematoxylin and eosin (H&E) staining was conducted according to standard procedures. Images were acquired using a DSZ2000 microscope (UOP Photoelectric Technology, Chongqing, China).

Primary antibodies included CA9 (Abclonal, A13682), HIF‐1α (Proteintech, 20960‐1‐AP), HIF‐1β (Proteintech, 14105‐1‐AP), HIF‐2α (Abclonal, A7553), CD70 (Abclonal, A2032), and PBRM1 (Abclonal, A0334). Technical support was provided by Biossci (Wuhan, China).

### 5.5 Immunofluorescence Staining

Fresh surgical specimens were fixed in formalin immediately after excision and embedded in paraffin. Multiplex immunofluorescence staining based on tyramide signal amplification (TSA) was performed on FFPE sections with technical support from Biossci (Wuhan, China).

After deparaffinization and rehydration, antigen retrieval was performed using citrate buffer (pH 6.0) or EDTA buffer (pH 9.0). Primary and secondary antibodies were applied sequentially, followed by TSA reagent incubation for 10 min. Antibody stripping was performed between staining cycles at 42 °C. Nuclei were counterstained with 4′,6‐diamidino‐2‐phenylindole (DAPI). Fluorescence images were captured using a DSZ2000 microscope. CD31 (Abclonal, A19014) was used for vascular endothelial cell identification. Detailed antibody information is provided above.

### 5.6 Small‐Animal PET/CT Imaging

Small‐animal PET/CT imaging was conducted using the Trans‐PET Discoverist 180 system (RAYCAN Technology, Suzhou, China). Tumor‐bearing mice were intravenously injected with 3.9–5.0 MBq of radiotracer and scanned at 30 min, 1 h, and 2 h post‐injection under anesthesia with 2% isoflurane. Each imaging session consisted of a 4‐min CT scan followed by a 10‐min PET acquisition. Images were reconstructed with attenuation correction. Regions of interest (ROIs) were quantified as percentage injected dose per gram of tissue (%ID/g) using Inveon Research Workplace software (Siemens, Germany).

### 5.7 Patient PET Imaging

^68^Ga‐DPI‐4452 PET/MR and ^18^F‐FDG PET/CT were performed on separate days within one week. For ^68^Ga‐DPI‐4452 imaging, patients underwent PET/MR scanning 30 min after tracer injection using a time‐of‐flight integrated PET/MR system (SIGNA PET/MR, GE Healthcare). Imaging duration was 15 min per bed position. The simultaneous MR protocol included T1‐weighted, T2‐weighted, fat‐suppressed T1 and T2 sequences, and diffusion‐weighted imaging.

^18^F‐FDG PET/CT was performed approximately 60 min after tracer administration using either a Discovery VCT scanner (GE Healthcare) or a Biograph Vision 600 system (Siemens Healthineers). Image fusion and analysis were performed on the Advantage Workstation (AW4.6, GE Healthcare). Images were independently reviewed by two nuclear medicine physicians with more than 5 years of experience. The readers were blinded to histopathological, molecular, and clinical outcome data during image quantification. Any discrepancies between the readers were resolved by consensus.

Regions of Interest (ROIs) were manually delineated on the fused PET/MR or PET/CT images with reference to the corresponding anatomical images. The SUVmax was automatically calculated by the workstation software, defined as the peak decay‐corrected tissue radioactivity concentration normalized by the injected dose and the patient's body weight. The tumor‐to‐background ratio (TBR) was subsequently calculated by dividing the tumor SUVmax by the corresponding reference background SUVmax, including blood pool, muscle, or renal parenchyma, as specified in the corresponding figure legends.

### 5.8 Western Blotting

Proteins were extracted from RCC tissues using RIPA lysis buffer supplemented with protease and phosphatase inhibitors. Equal amounts of protein (30 µg per lane) were separated by sodium dodecyl sulfate‐polyacrylamide gel electrophoresis (SDS‐PAGE) and transferred to polyvinylidene fluoride (PVDF) membranes. After blocking, membranes were incubated with primary antibodies against CA9 (Abclonal, A13682), PBRM1 (Abclonal, A0334), and GAPDH (Abclonal, AC002), followed by HRP‐conjugated secondary antibodies. Signals were detected using standard chemiluminescence methods.

### 5.9 Transcriptomic and Metabolomic Profiling

Fresh surgical specimens were snap‐frozen in liquid nitrogen immediately after excision. Bulk RNA‐seq was performed by BGI Genomics (Shenzhen, China). Untargeted metabolomic profiling was conducted by Metware Biotechnology (Wuhan, China) according to standard protocols.

### 5.10 Patient‐Derived Xenograft (PDX) Models

Four‐week‐old NCG mice were obtained from GEMPharmatech (Nanjing, China) and allowed to acclimatize for 1 week before tumor implantation. Fresh tumor tissues were implanted subcutaneously into approximately 5‐week‐old NCG mice within 2 h after surgical resection. Tumors were passaged to the third generation before therapeutic experiments. When tumor volume exceeded 70 mm^3^, mice were treated with axitinib (25 mg/kg, once daily, MedChemExpress). Tumor size and body weight were measured every 3 days. Tumor volume was calculated as (length × width^2^)/2. Mice were monitored regularly for general health status and signs of distress throughout the experiment. Humane endpoints were predefined as tumor volume exceeding 1000 mm^3^, body weight loss greater than 15% from baseline, tumor ulceration, necrosis, or infection, impaired mobility, severe lethargy or signs of distress, inability to access food or water, or any other condition requiring euthanasia based on veterinary assessment. Mice reaching humane endpoints were euthanized and recorded as events in the survival analysis. Survival time was defined as the time from treatment initiation to euthanasia due to reaching predefined humane endpoints.

### 5.11 Public Data Acquisition and Bioinformatic Analysis

Bulk transcriptomic data for RCC were obtained from The Cancer Genome Atlas (TCGA), including the TCGA‐KIRC, TCGA‐KIRP, and TCGA‐KICH cohorts. Normal tissue expression data (transcripts per million, TPM) were retrieved from the GTEx database, and proteomic data were obtained from the CPTAC dataset (PDC000127). Plasma membrane–associated genes were curated from the Molecular Signatures Database using the gene set GOCC_PLASMA_MEMBRANE_REGION (Table S2).

Public scRNA‐seq datasets (SRZ190804 [26], SCP1288 [27], GSE210038 [28], and GSE159115 [29]) were analyzed using Seurat in R (v4.2.0). After quality control, normalization, and identification of highly variable genes, datasets were integrated to correct for batch effects, followed by graph‐based clustering and Uniform Manifold Approximation and Projection (UMAP) visualization. Cell types were annotated based on canonical marker genes.

Malignant tumor cells were identified by inferring large‐scale CNVs using inferCNV, with B cells serving as non‐malignant reference controls. Tumor cells were stratified into CA9‐high and CA9‐low groups based on relative CA9 expression. Tumor cell stemness was assessed using CytoTRACE2, and stemness scores were visualized on UMAP embeddings and compared between groups using two‐sided Student's t tests.

Publicly available spatial transcriptomic data were obtained from the Gene Expression Omnibus dataset GSE175540 [23]. Two samples, GSM5924052 and GSM5924046, were included in the present analysis. The spatial gene‐expression matrices and corresponding spatial coordinate information were downloaded from the GEO repository and reanalyzed using Scanpy.

Angiogenesis pathway activity was quantified using scanpy.tl.score_genes based on an angiogenesis‐related gene set obtained from the Molecular Signatures Database. The association between spatial CA9 expression and angiogenesis scores was evaluated using Pearson correlation analysis. Spatial expression and pathway‐score distributions were visualized according to the spatial coordinates of individual spots.

### 5.12 Statistical Analysis

All statistical analyses were performed using R (v4.2.0). Unless otherwise specified, statistical details including sample size, data presentation, and statistical tests are provided in the corresponding figure legends, tables, or text. Categorical variables are presented as numbers and percentages. Data distribution was assessed for normality before statistical testing. Continuous variables between two groups were compared using two‐sided Student's *t* tests when the data followed a normal distribution or when normality could be reasonably assumed. Otherwise, the Mann‐Whitney U test was applied. For comparisons among more than two groups, one‐way analysis of variance followed by appropriate post hoc tests was used for normally distributed data, whereas the Kruskal–Wallis test followed by post hoc pairwise comparisons was used for non‐normally distributed data, where applicable. Categorical variables were compared using χ^2^ or Fisher's exact tests, as appropriate. Logistic regression analysis was performed to estimate odds ratios and corresponding 95% confidence intervals (CI). Correlation analyses were conducted using Pearson or Spearman correlation coefficients, depending on data distribution. Survival analyses were performed using the Kaplan‐Meier method and compared by log‐rank tests. All tests were two‐sided unless otherwise specified, and *P* values < 0.05 were considered statistically significant.

### 5.13 Ethics Approval and Consent to Participate

All animal procedures were approved by the Animal Ethics Committee of Huazhong University of Science and Technology (Approval No. [2024]‐5198). The clinical study protocol was approved by the Ethics Committee of Union Hospital, Tongji Medical College, Huazhong University of Science and Technology (Approval No. [2023]‐0847). Written informed consent was obtained from all participants prior to surgery.

## Author Contributions

X.Z., K.W., and D.J conceived and supervised the project. KC and SC designed the experiments. K.C., S.C., R.L., J.S., Y.W., X.Z., X.S., Z.W., and QW performed all experiments. L.L., X.L., Z.D., H.Y., and YL were in charge of patient enrollment and data collection. All authors analyzed and discussed the data. K.C., S.C., and R.L wrote the paper.

## Funding

This work was supported by the National Natural Science Foundation of China (Grant no. 82573418, 82303033, 82473436, 8250103906, 22277031), Shenzhen Medical Research Funds (Grant no. B2302054), Natural Science Foundation of Wuhan (Grant no. 2024040801020349), Shenzhen Science and Technology Program (JCYJ20250604191049062), the Open Research Fund of Hubei Province Key Laboratory of Precision Radiation Oncology (No. 2025ZLJZFL011), Hubei Science and Technology Innovation Talent Program (2023DJC162), Wuhan Union Hospital Clinical Translation Program (2025‐XHCGZHJJ‐012)

## Conflicts of Interest

The authors have declared that no conflicts of interest.

## Supporting information




**Supporting File 1**: advs76622‐sup‐0001‐SuppMat.docx.

**Supporting File 2**: advs76622‐sup‐0002‐TableS1.xlsx.

**Supporting File 3**: advs76622‐sup‐0003‐TableS2.xlsx.

**Supporting File 4**: advs76622‐sup‐0004‐TableS3.xlsx.

**Supporting File 5**: advs76622‐sup‐0005‐blotts.zip.

## Data Availability

All publicly available datasets used in this study are listed in the corresponding *Methods* sections. Other data supporting the findings of this study are available from the corresponding author upon reasonable request.
