# Supplementary material for: CA9‐Targeted PET Imaging for Noninvasive Discrimination of Clear Cell Renal Cell Carcinoma and Associated Tumor Biological Features
Source: Adv Sci (Weinh). 2026 Jul 20:e76622. Online ahead of print. doi: 10.1002/advs.76622 (PMC13383157; doi:10.1002/advs.76622)
Supplement: Supplementary file 1 — Supporting File 1: advs76622‐sup‐0001‐SuppMat.docx. [file ADVS-9999-e76622-s001.docx]

**Supplementary Method**

**Metabolomic profiling**

Fresh surgical specimens were snap-frozen in liquid nitrogen immediately after excision. Untargeted metabolomic profiling was conducted by Metware Biotechnology (Wuhan, China) according to standard protocols. Liquid chromatography coupled with tandem mass spectrometry (LC-MS/MS) was employed to enable comprehensive metabolite separation and identification. Metabolomics analysis aims to systematically detect and quantify metabolites with biological relevance and statistical significance from complex biological samples, thereby elucidating metabolic states and regulatory alterations. The overall workflow comprised experimental acquisition and downstream data analysis, including differential metabolite screening and pathway enrichment. Based on standardized experimental design, sample collection and processing, metabolite extraction, and LC-MS/MS acquisition, raw metabolomic data were subjected to metabolite identification, quality control, and statistical filtering to enable functional interpretation of metabolic alterations across samples.

For tissue-based metabolomics, frozen specimens were retrieved from -80 °C storage and thawed on ice until amenable to sectioning, with all subsequent procedures performed under cold conditions. Tissues were finely minced, homogenized, and approximately 20 mg (±1 mg) of each sample was accurately weighed into pre-labeled centrifuge tubes. A stainless steel bead was added to each tube, followed by mechanical homogenization at 30 Hz for 20 s. Samples were briefly centrifuged at 4 °C to collect material at the tube bottom, after which 400 µL of 70% methanol aqueous extraction solvent containing internal standards was added. The mixture was vortexed at 1500 rpm for 5 min and incubated on ice for 15 min to ensure efficient metabolite extraction. Subsequently, samples were centrifuged at 12,000 rpm for 10 min at 4 °C, and 300 µL of the supernatant was transferred to a fresh tube and incubated at -20 °C for 30 min to precipitate residual proteins. After a second centrifugation at 12,000 rpm for 3 min at 4 °C, 200 µL of the clarified supernatant was collected into autosampler vials for LC-MS/MS analysis.

Chromatographic separation was performed using a Waters ACQUITY Premier HSS T3 column (1.8 µm, 2.1 mm × 100 mm). The mobile phase consisted of solvent A (0.1% formic acid in water) and solvent B (0.1% formic acid in acetonitrile). The column temperature was maintained at 40 °C with a flow rate of 0.4 mL/min, and the injection volume was set to 3 µL.

Raw mass spectrometry data were converted to mzML format using ProteoWizard and processed with the XCMS pipeline for peak detection, retention time alignment, and peak integration. Metabolic features with a missing rate greater than 50% across samples were excluded. Missing values were imputed using a combined strategy, in which features with blank values exceeding 50% were filled with one-fifth of the minimum detected value, whereas features with lower missing rates were imputed using a k-nearest neighbor (KNN) algorithm. Signal drift and batch effects were corrected using support vector regression (SVR). Metabolite annotation was achieved by matching corrected features against an in-house Metware database, supplemented by integrated public databases and predictive libraries. Only metabolites with a composite identification score greater than 0.5 and a coefficient of variation (CV) below 0.3 in quality control samples were retained. Data from positive and negative ionization modes were subsequently merged, and in cases of duplicate annotations, metabolites with the highest confidence level and identification score were selected for downstream analyses.

**Transcriptomic profiling**

Fresh surgical specimens were snap-frozen in liquid nitrogen immediately after excision. Bulk RNA sequencing was performed by BGI Genomics (Shenzhen, China). Total RNA was extracted from tissue or cell samples using appropriate extraction methods depending on sample type, followed by rigorous quality control assessment. RNA integrity, purity, and concentration were evaluated prior to library construction, and only samples meeting quality criteria were subjected to downstream sequencing.

For eukaryotic samples, messenger RNA was enriched from total RNA via oligo(dT) selection based on the presence of poly(A) tails. Purified mRNA was fragmented into short fragments using divalent cations under elevated temperature. Fragmented RNA was then reverse-transcribed into first-strand cDNA using random hexamer primers, followed by second-strand cDNA synthesis to generate double-stranded cDNA. The resulting cDNA fragments were purified, end-repaired, A-tailed, and ligated to sequencing adapters. Adapter-ligated products were size-selected to obtain fragments of the desired length, followed by PCR amplification and purification to generate the final sequencing libraries. Library quality was assessed by measuring fragment size distribution and library concentration prior to sequencing.

Qualified libraries were pooled according to effective concentration and target sequencing depth and subjected to high-throughput sequencing on an Illumina platform using a paired-end 150 bp (PE150) strategy. Sequencing was performed based on sequencing-by-synthesis chemistry, in which fluorescently labeled nucleotides were incorporated into nascent DNA strands during cluster amplification on a flow cell. Fluorescence signals emitted upon nucleotide incorporation were captured and converted into base calls by the instrument software, generating raw sequence reads for downstream analysis.

**Supplementary Figures**


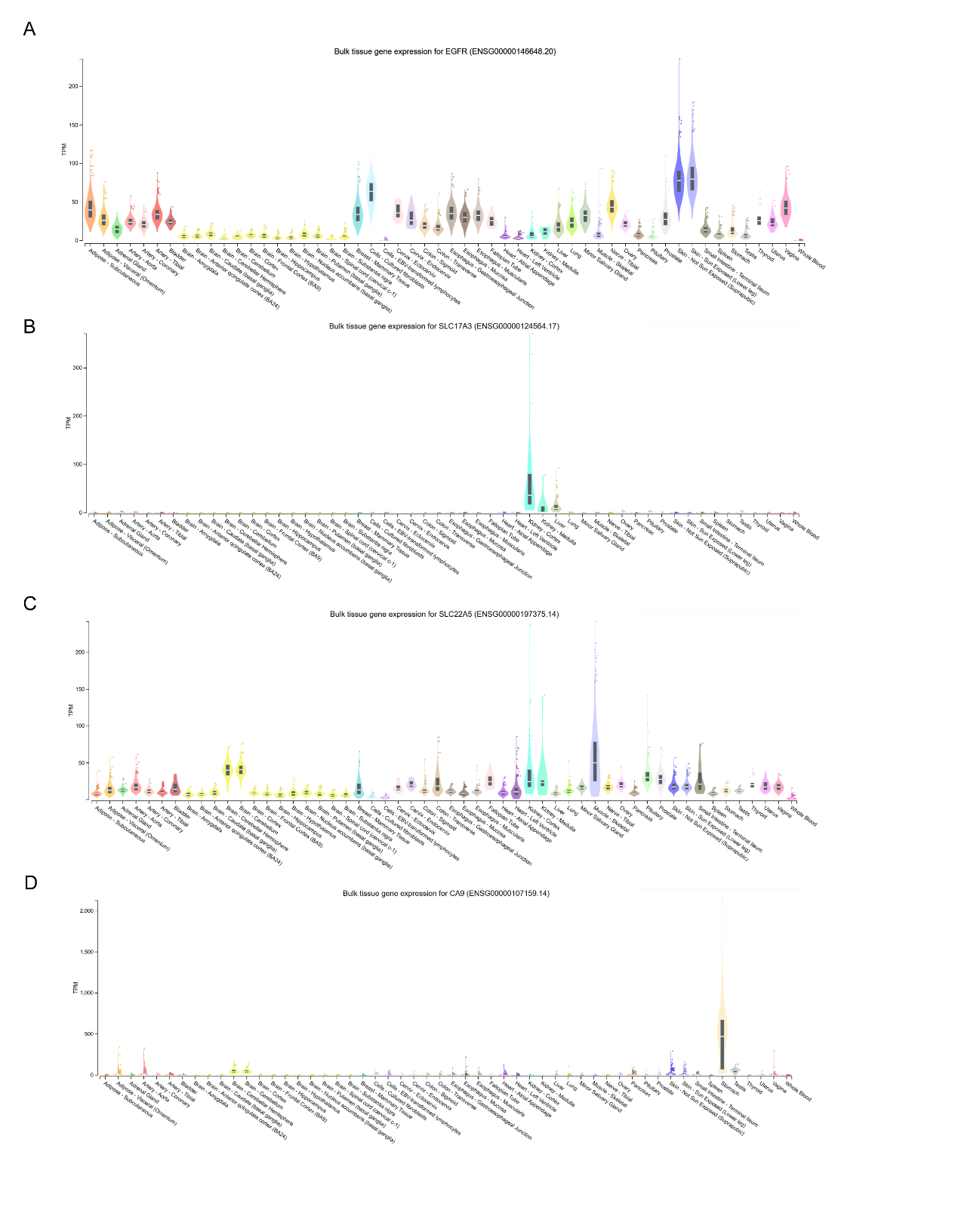


**Figure S1 Bulk tissure gene expression for candidate genes.** **(A)** Bulk tissure gene expression for EGFR. **(B)** Bulk tissure gene expression for SLC17A3. **(C)** Bulk tissure gene expression for SLC22A5. **(D)** Bulk tissure gene expression for CA9.


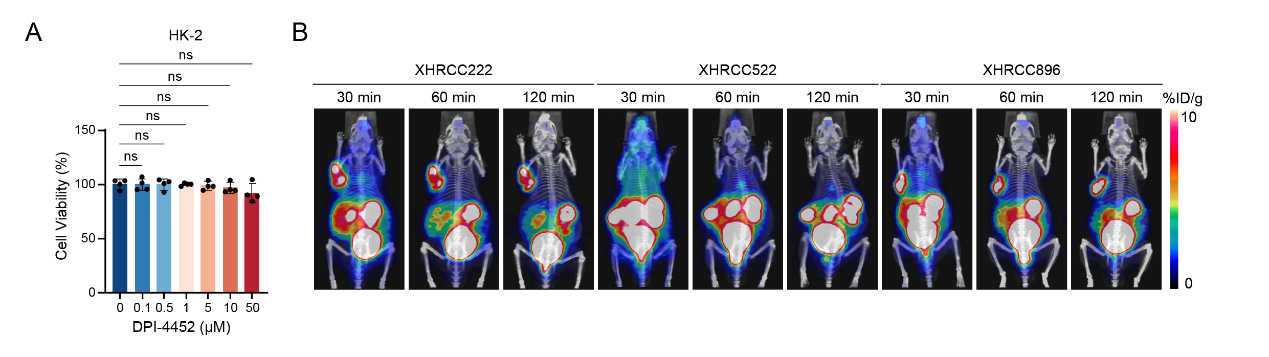


**Figure S2** Safety evaluation and uptake characterization of the CA9-targeted radiotracer. **(A)** Effect of different concentrations of DPI-4452 on the viability of HK-2 cells. (One-way ANOVA test). **(B)** Representative longitudinal CA9-targeted PET images of ccRCC patient-derived xenograft (PDX) models acquired at 30, 60, and 120 minutes after intravenous injection of a CA9-targeted radiotracer (⁶⁸Ga-DPI-4452).


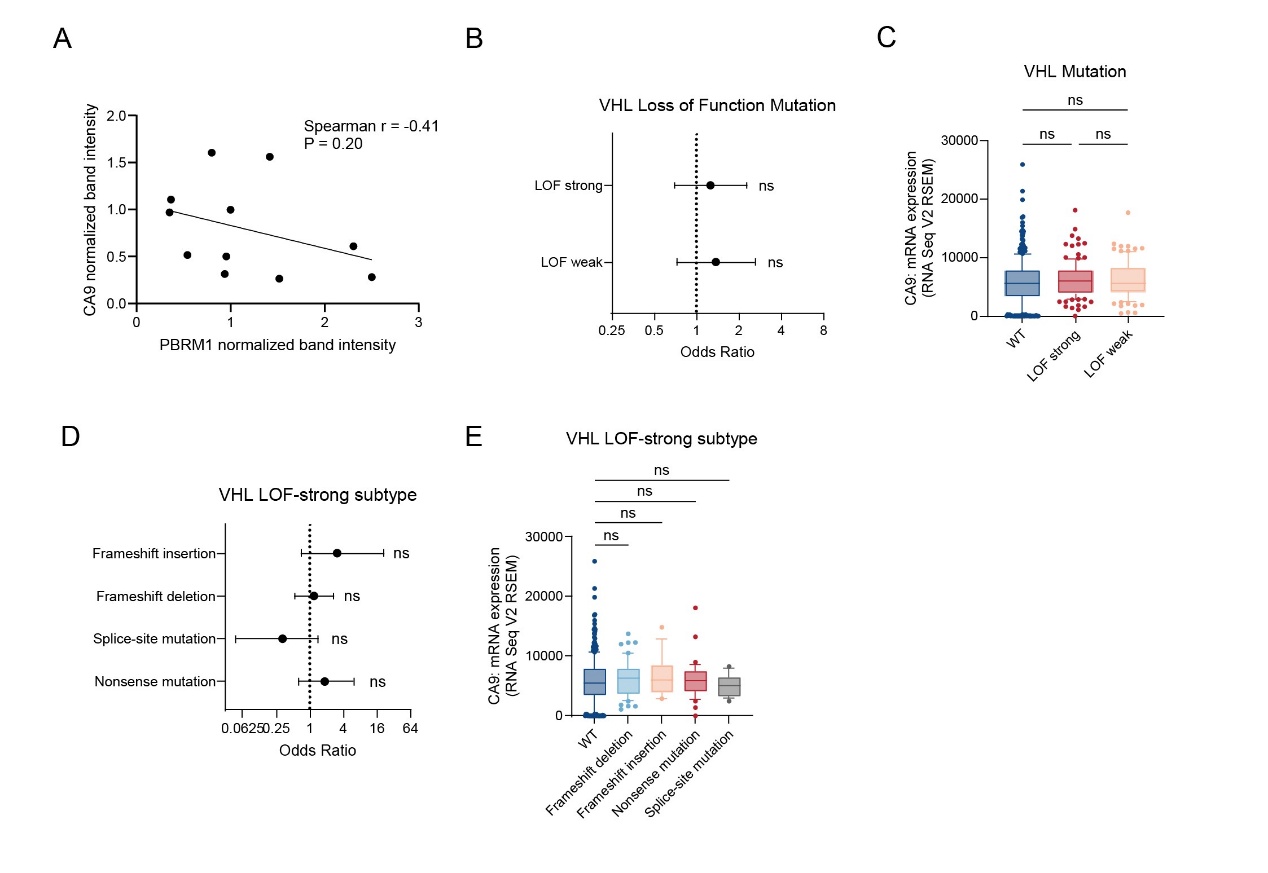


**Figure S3.** **Association of CA9 expression with PBRM1 protein expression and VHL alterations in ccRCC. (A)** Densitometric correlation analysis of CA9 and PBRM1 protein expression in ccRCC tissue specimens based on the western blot shown in Figure 5I. Relative CA9 and PBRM1 band intensities were quantified by densitometric analysis and normalized to GAPDH (n = 11). **(B)** Odds ratios comparing the frequencies of VHL loss-of-function (LOF) strong and LOF weak mutations between CA9-high and CA9-low ccRCC tumors, analyzed using logistic regression. **(C)** Differences in CA9 mRNA expression among ccRCC tumors harboring different VHL mutation types. Kruskal-Wallis test with Dunn’s multiple comparisons test (n = 533). **(D)** Odds ratios comparing the frequencies of distinct VHL LOF strong mutation subtypes between CA9-high and CA9-low ccRCC tumors, assessed using logistic regression. **(E)** Comparison of CA9 mRNA expression levels among ccRCC tumors with different VHL LOF strong mutation subtypes. Kruskal-Wallis test with Dunn’s multiple comparisons test (n = 533). Stratification criteria: for TCGA-KIRC mutation analyses in panels B and D, CA9-high and CA9-low tumors were defined as the upper and lower 25% of CA9 TPM expression among samples with available mutation data.


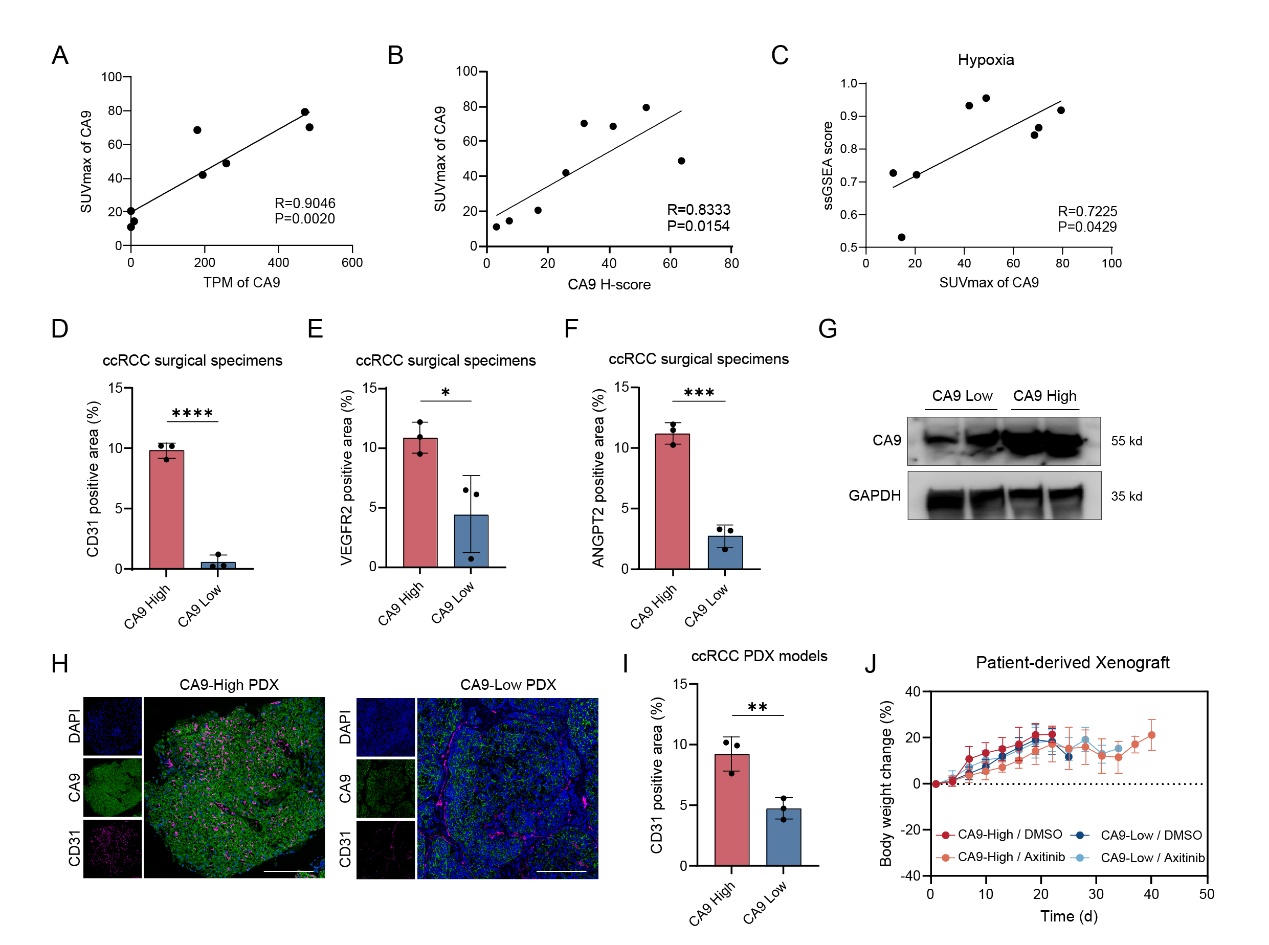


**Figure S4. Validation of CA9-targeted PET uptake, angiogenic features, and PDX model monitoring. (A)** Correlation analysis between CA9-targeted PET SUVmax values and TPM of CA9 in RNA-seq of ccRCCs. (Spearman correlation, n = 8). **(B)** Correlation analysis between CA9-targeted PET SUVmax values and H-score of CA9 in ccRCCs. (Spearman correlation, n = 8). **(C)** Correlation analysis between CA9 PET SUVmax values and Hallmark Hypoxia scores. (Spearman correlation, n = 8). **(D-F)** Quantification of the percentage of CD31-positive area (D), VEGFR2-positive area (E), ANGPT2-positive area (F) and in immunofluorescence-stained surgical specimens from CA9-high and CA9-low ccRCCs (n = 3 for each group). Quantification was performed using ImageJ. For each specimen, at least three representative fields were analyzed and averaged. Data were compared using unpaired two-tailed Student’s t-tests. **(G)** Validation of CA9 expression in ccRCC PDX models. Representative images from three independent experiments are shown. **(H)** Representative immunofluorescent staining of CD31 and CA9 in CA9-high and CA9-low ccRCC PDX tumors (scale bar = 200 μm). **(I)** Quantification of the percentage of CD31-positive area in immunofluorescence-stained ccRCC PDX tumors with high and low CA9 expression (n = 3 for each group). Quantification was performed using ImageJ. For each specimen, at least three representative fields were analyzed and averaged. Data were compared using unpaired two-tailed Student’s t-tests. **(J)** Longitudinal body weight changes of mice bearing ccRCC PDX tumors during the monitoring period (n = 6 for each group).
