# Supplementary material for: CA9‐Targeted PET Imaging for Noninvasive Discrimination of Clear Cell Renal Cell Carcinoma and Associated Tumor Biological Features
Source: Adv Sci (Weinh). 2026 Jul 20:e76622. Online ahead of print. doi: 10.1002/advs.76622 (PMC13383157; doi:10.1002/advs.76622)
Supplement: Supplementary file 5 — Supporting File 5: advs76622‐sup‐0005‐blotts.zip. [file ADVS-9999-e76622-s004.zip › advs76622-sup-0005-blotts/Original WB blot.pptx]

## Slide 1
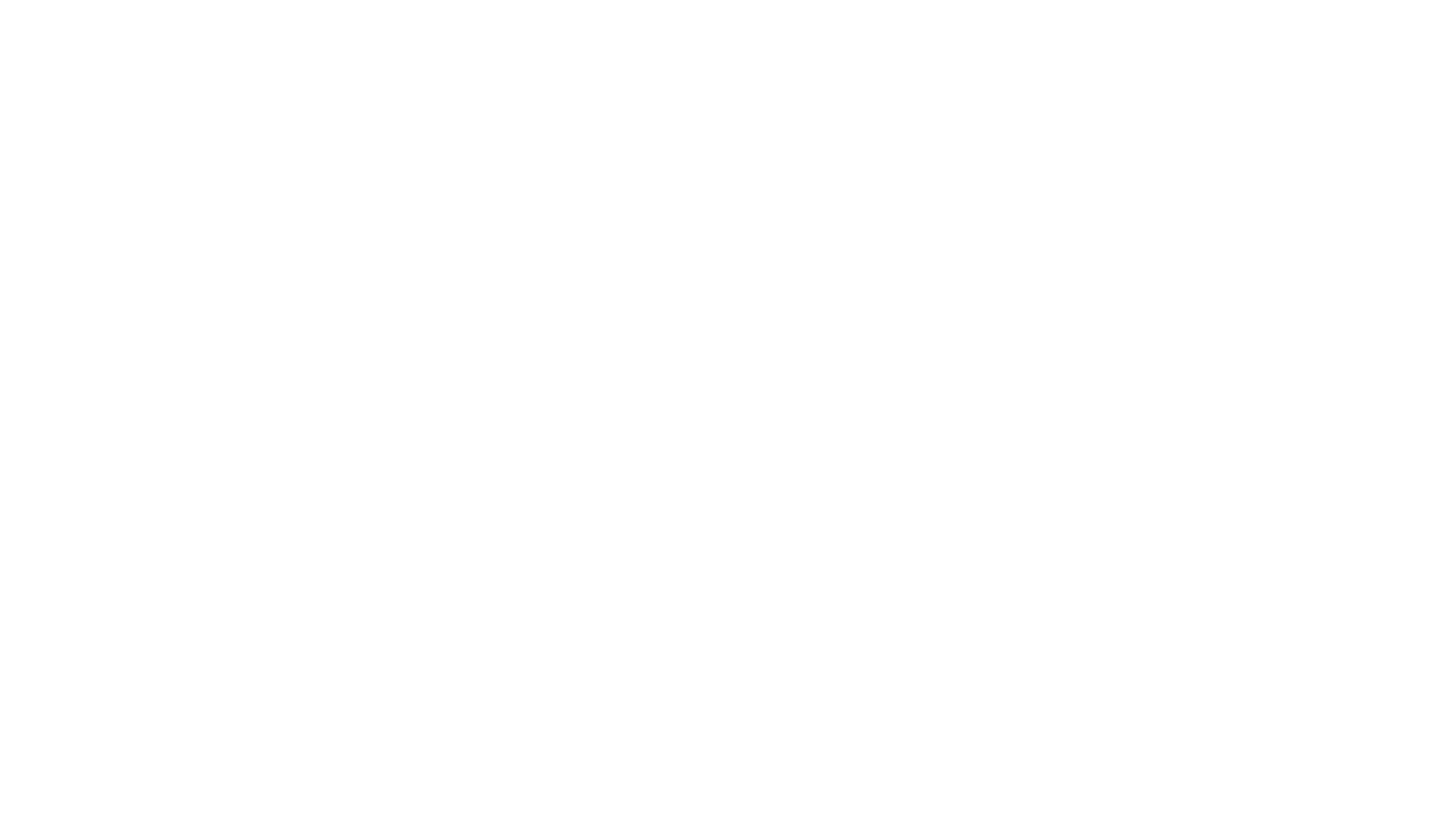

## Slide 2
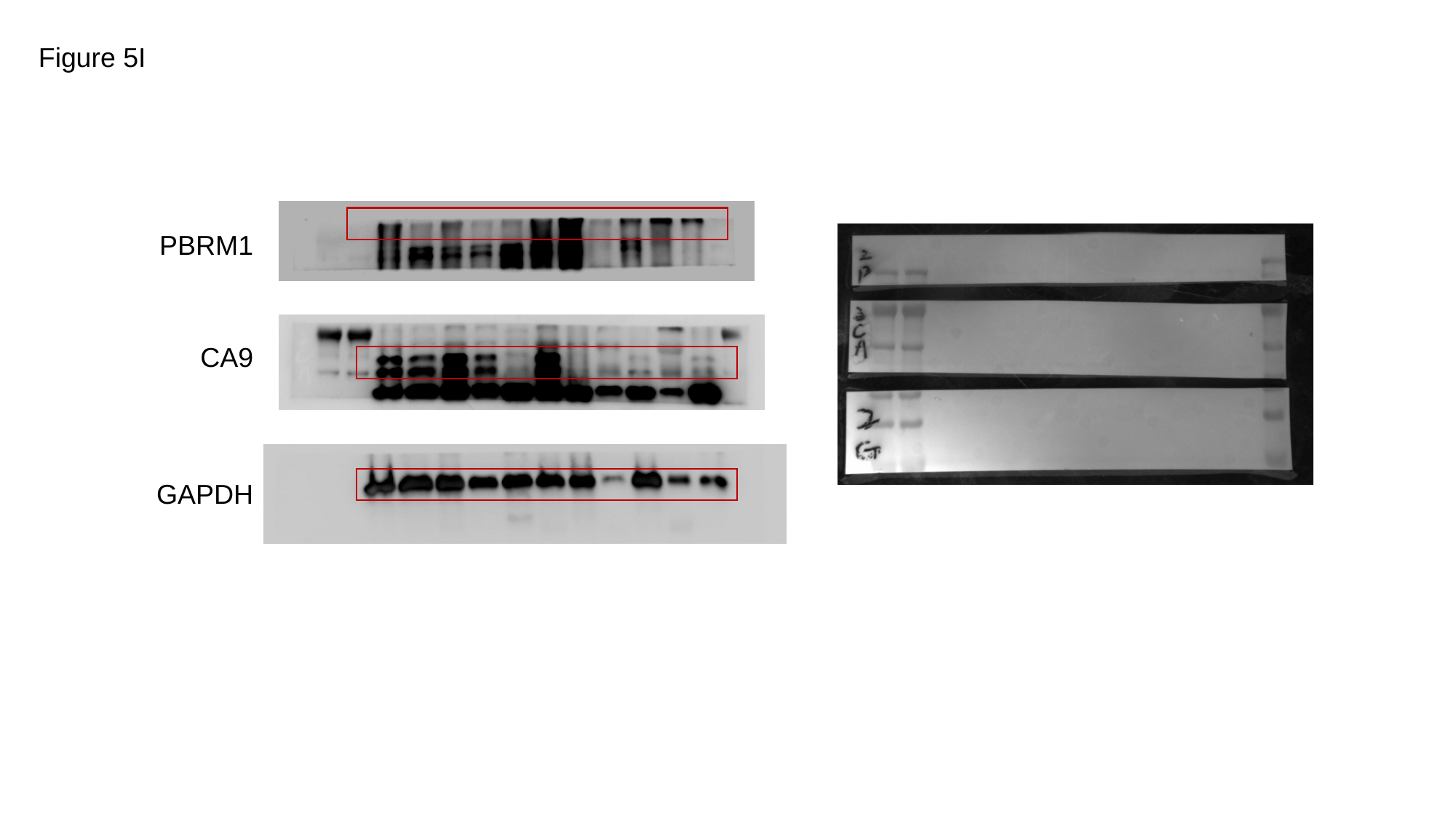

Figure 5I
PBRM1
CA9
GAPDH

## Slide 3
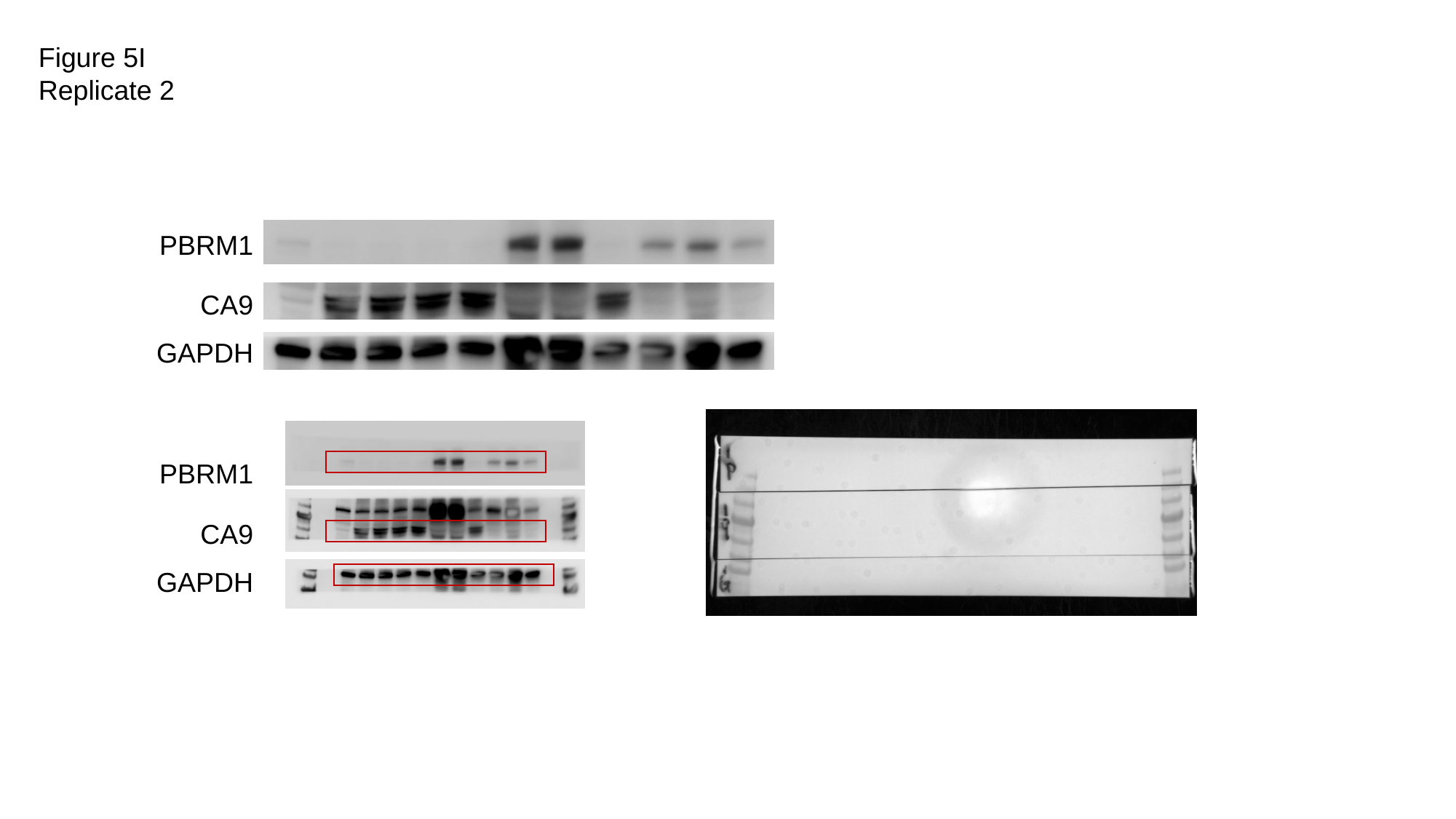

Figure 5I Replicate 2
PBRM1
CA9
GAPDH
PBRM1
CA9
GAPDH

## Slide 4
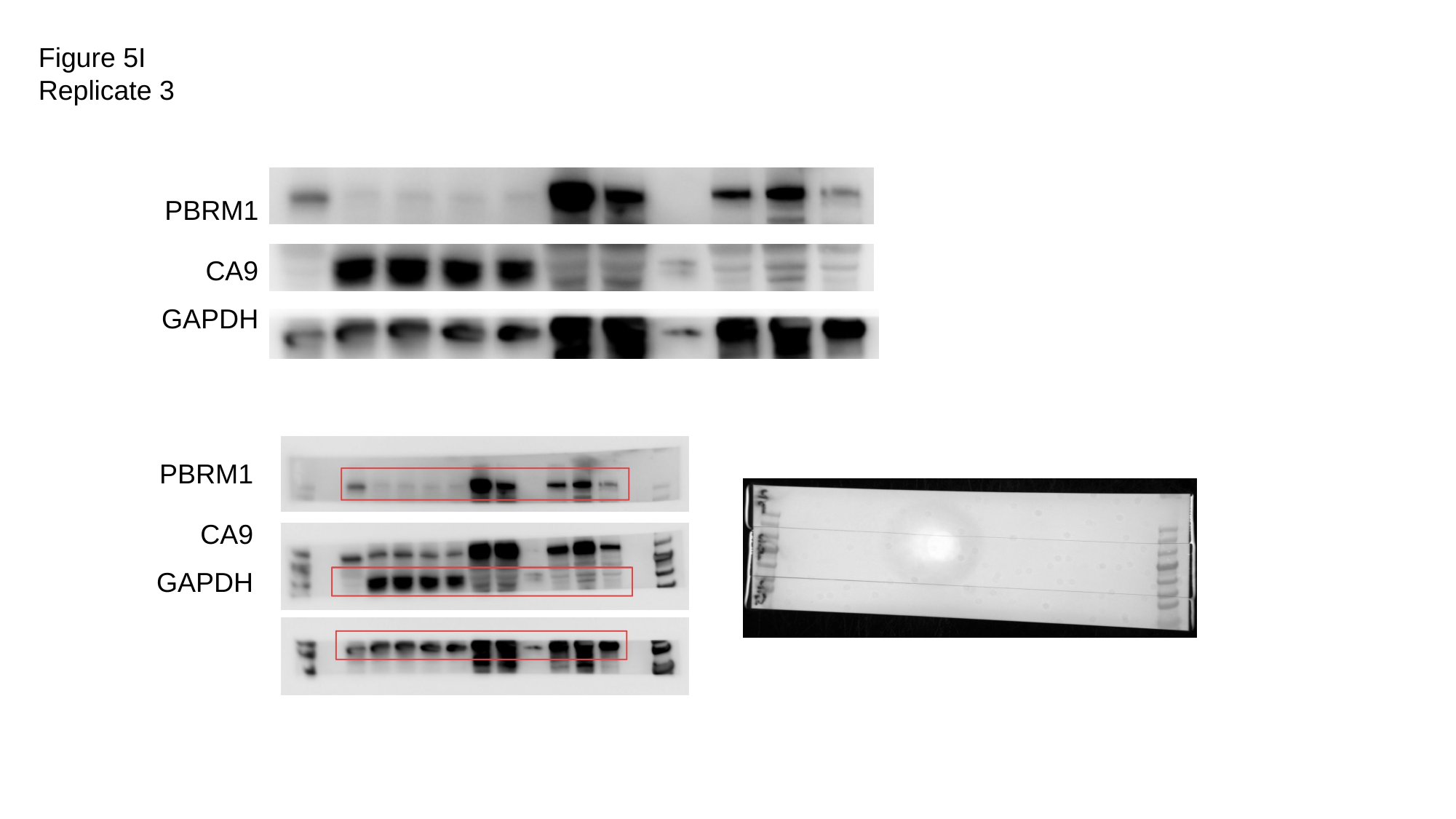

Figure 5I Replicate 3
PBRM1
CA9
GAPDH
PBRM1
CA9
GAPDH

## Slide 5
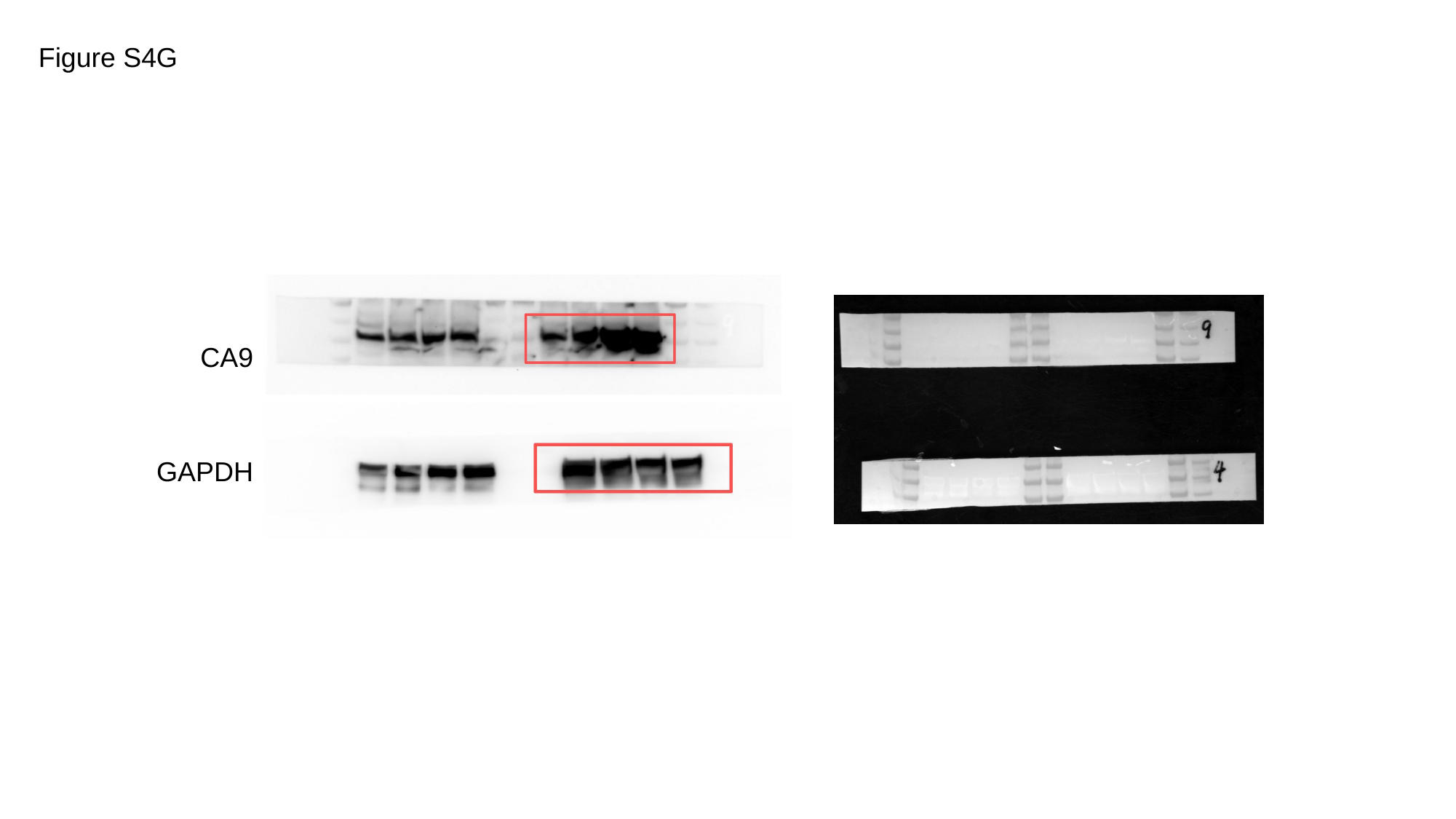

Figure S4G
CA9
GAPDH

## Slide 6
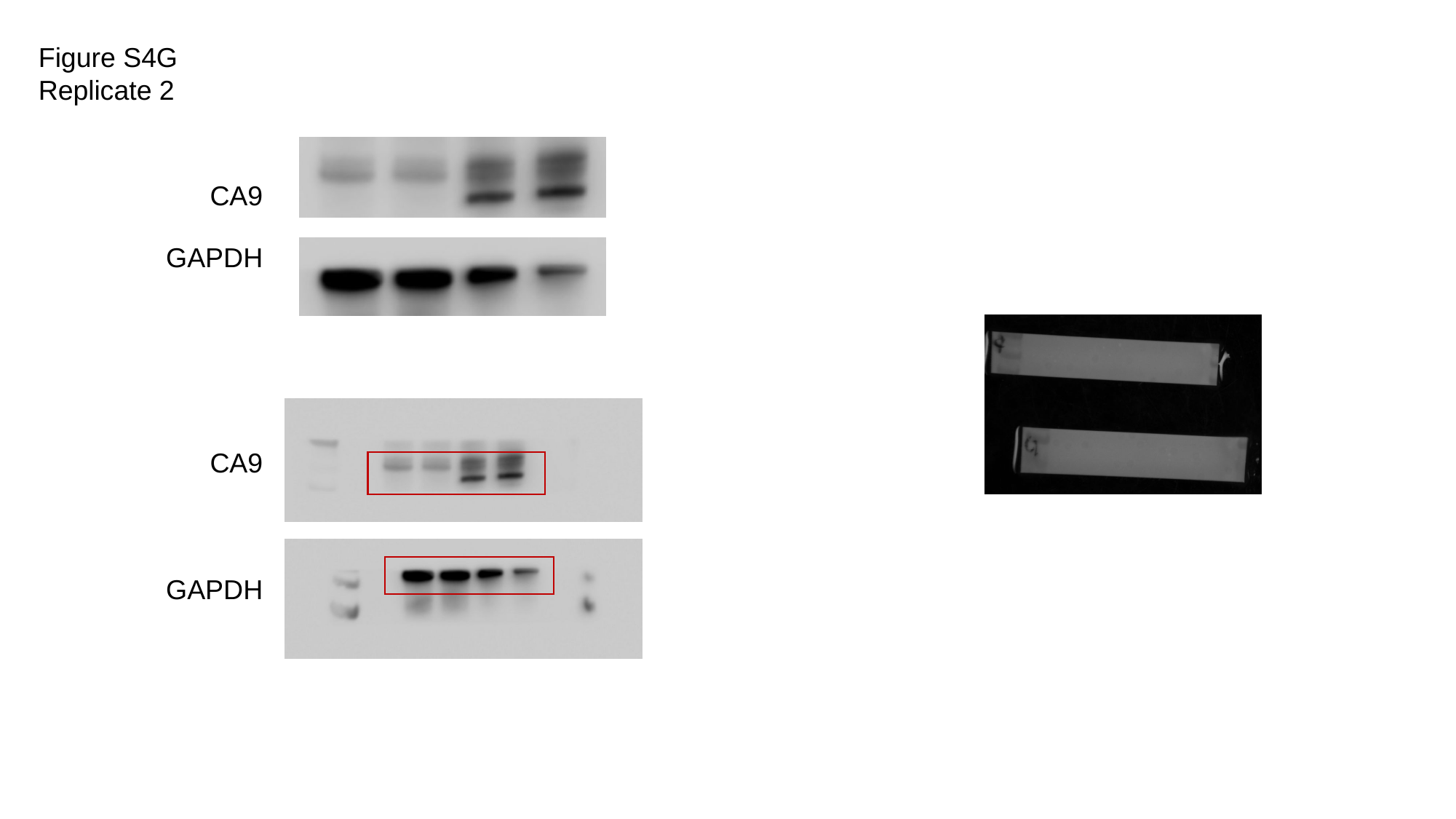

Figure S4G
Replicate 2
CA9
GAPDH
CA9
GAPDH

## Slide 7
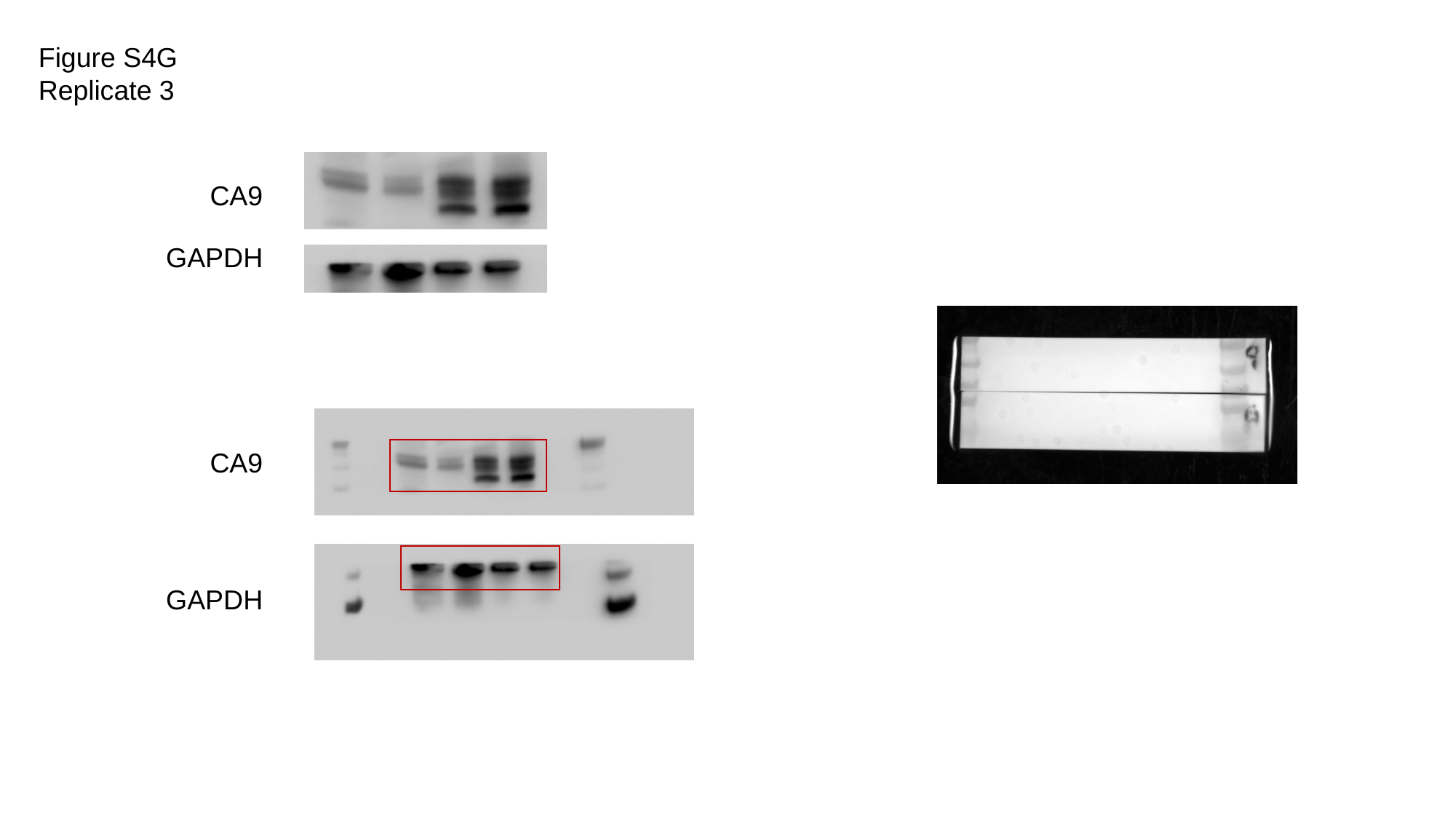

Figure S4G
Replicate 3
CA9
GAPDH
CA9
GAPDH
